# Dual‐Modal Dielectric Elastomer System for Simultaneous Energy Harvesting and Actuation

**DOI:** 10.1002/advs.202410724

**Published:** 2024-12-11

**Authors:** Zhiyuan Zhang, Wenwei Huang, Shaodi Zheng, Jianbo Tan, Jinzhan Cheng, Jiancheng Cai, Shiju E, Zisheng Xu

**Affiliations:** ^1^ Key Laboratory of Urban Rail Transit Intelligent Operation and Maintenance Technology & Equipment of Zhejiang Province College of Engineering Zhejiang Normal University Jinhua 321004 P. R. China; ^2^ Jinhua Intelligent Manufacturing Research Institute Jinhua 321004 P. R. China

**Keywords:** actuator, dielectric elastomer, electromechanical instabilities, generator

## Abstract

Dielectric elastomers (DEs) have promising capabilities for soft electromechanical systems, including those for actuation and energy generation. However, their widespread application is restricted by electromechanical instability (EMI) and the requirement for high‐voltage operation. This study presents a dual‐modal DE system that effectively overcomes these limitations by leveraging a dual‐membrane structure. The proposed structure not only suppresses EMI through charge sharing but also enables simultaneous energy harvesting and actuation, enhancing the overall electrical performance of the system. The system demonstrated a remarkable improvement in output performance, exceeding that of traditional single‐modal DE generators by up to 30%. The practicality of the system is developed by integrating it into a mechanically powered soft robot capable of locomotion and environmental monitoring using a wireless temperature sensor. This study paves the way for the development of advanced DE‐based systems with enhanced stability, functionality, and potential for diverse applications in soft robotics, energy harvesting, and other areas that require coupled electromechanical capabilities.

## Graphical abstract

A novel dual‐membrane strategy is presented to simultaneously address electromechanical instability (EMI) and enable energy harvesting and actuation in dielectric elastomer (DE) systems. This approach, implemented in a dual‐modal DE system, enhances actuation performance, and increases electrical output by 30% compared to traditional single‐modal DE generators, advancing DE technology for soft robotics and energy harvesting applications.

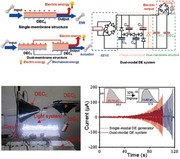

## 1 Introduction

The field of soft electromechanical systems has witnessed a surge in research and development, particularly in energy harvesting and robotics.^[^ ^1^, ^2^, ^3^, ^4^ ^]^ Dielectric elastomers (DEs) have emerged as promising candidates for electromechanical transducers owing to their inherent advantages, such as structural simplicity, high efficiency, and ease of control.^[^ ^5^, ^6^, ^7^ ^]^ Functioning on the principle of a variable capacitor,^[^ ^8^ ^]^ DE transducers enable the conversion of electrical energy into mechanical work (actuator)^[^ ^9^, ^10^ ^]^ and vice versa (generator).^[^ ^11^, ^12^, ^13^, ^14^ ^]^ These transducers typically consist of a DE film sandwiched between compliant electrodes to form a dielectric elastomer capacitor (DEC). However, two key factors impede the wide usage of DE transducers: the requirement for a high‐voltage source and their susceptibility to electromechanical instability (EMI). A high voltage can be achieved using a voltage multiplier or charge pump circuits.^[^ ^15^, ^16^, ^17^ ^]^ Although solutions for generating high voltage exist, EMI remains a significant hurdle, limiting the performance and reliability of these devices.

EMI occurs when the electrostatic force between the DE electrodes surpasses the elastic restoring force of the material. This force imbalance is particularly pronounced during high‐voltage operations, where the DE films undergo significant strain, especially during the release phase.^[^ ^18^, ^19^, ^20^, ^21^ ^]^ Consequently, the localized regions experience an increase in charge density, and the electric field is subsequently, intensified. This cascade effect leads to progressively stronger electrostatic forces and further strain accumulation, ultimately causing a rapid reduction in thickness and a corresponding decrease in the capacitance change. Consequently, potential damages, such as electric breakdown, hysteresis,^[^ ^22^ ^]^ fatigue,^[^ ^21^ ^]^ and unstable snap‐through behavior,^[^ ^23^, ^24^ ^]^ may cause catastrophic failure of the device. Efforts have been made in research and engineering aimed at understanding, modeling, and mitigating EMI to enhance the performance and durability of DEs. Current solutions include two primary approaches: applying a constant strain to the DE materials and developing DE materials with enhanced properties. The former approach utilizes techniques, such as pre‐stretching,^[^ ^25^, ^26^ ^]^ rigid constraints,^[^ ^27^, ^28^ ^]^ and a special loading path,^[^ ^29^ ^]^ that effectively prevent strain distribution and the onset of EMI. Regarding material development, numerous design strategies,^[^ ^30^ ^]^ including interpenetrating networks,^[^ ^31^ ^]^ bottle‐brush structures,^[^ ^32^ ^]^ bimodal‐networks,^[^ ^33^ ^]^ and high‐dielectric constant architectures,^[^ ^34^ ^]^ have been explored to fabricate elastomers with desirable rapid strain‐hardening behavior, thus eliminating or suppressing EMI. However, these two methods introduce additional issues, such as a relatively low maximum strain and weak mechanical strength, which affect the performance of electromechanical energy conversion.

**Figure 1:**
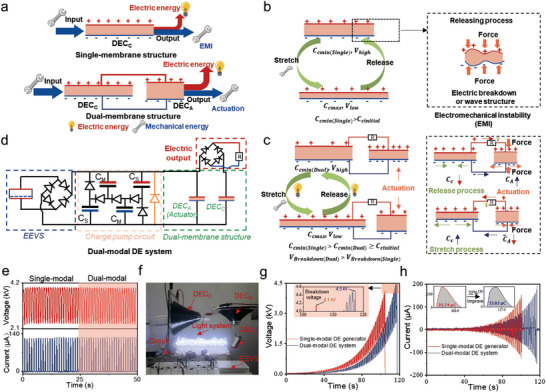
Structural design and operating mechanism of the dual‐modal DE system. a) Energy conversion behaviors of single‐membrane and dual‐membrane structures. b) EMIs of the single‐membrane structure. c) Working schematic of the dual‐membrane structure. d) Circuit diagrams of the dual‐modal DE system. e) Electric performance contrast between the single‐modal DE generator and dual‐modal DE system under the saturating voltage of 4 kV. f) Electric output and actuation behavior of the dual‐modal DE system. g,h) Comparison of electrical performance of single‐modal DE generator and dual‐modal DE system. g) Breakdown voltage. h) Electric current. (Frequency: 0.8 Hz, the saturating voltage: 4 kV; DEC_C_’s capacitance varied from 4.2 to 12.7 nF).

In this study, we developed a dual‐membrane strategy to suppress EMI and constructed a dual‐modal DE system that simultaneously realizes energy harvesting and actuation. The dual‐membrane structure incorporates a secondary DEC, denoted as DEC_A_, to share charges from the primary DEC, denoted as DEC_C_, via charge transfer. This approach effectively suppresses EMI while simultaneously enhancing energy output and enabling actuation capabilities (**Figure** **1**a). A detailed investigation of the behavior of the transfer charge in the dual‐modal DE system indicated the relationship among the actuation performance, charge transfers within the system, and charge transfers within the dual‐membrane structure. The actuation performance of the DEC_A_ stems from the charge‐driven capacitance variation, which depends on the electric energy storage and mechanical strain capacity. The dual‐modal DE system with a dual‐membrane structure exhibited a significant improvement in electrical output performance, with the both peak power and average power exceeding those of the traditional single‐modal DE generator by over 30%. To demonstrate the practical application of this technology, we successfully implemented a dual‐modal DE system in a mechanically powered robot capable of efficiently converting the input mechanical energy into both electrical and mechanical energy. Our findings highlight the dual‐membrane structure as a promising approach for overcoming the limitations of conventional DE systems, paving the way for advancements in soft robotics, energy harvesting, and other related fields.

## 2 Results

### 2.1 Structural Design and Operating Mechanism of the Dual‐Modal DE System

Figure 1a presents the contrasting design concepts of dual‐membrane and traditional single‐membrane structures. Both structures can convert the external mechanical energy into electrical energy based on the principle of variable capacitance. The traditional design employs a single‐membrane DEC, denoted as a DEC_C_, consisting of a DE membrane sandwiched between compliant electrodes. Traditional DE devices, which have a single‐membrane structure, suffer from EMI owning to instability between the electrostatic stress and elastic stress during the high‐voltage stage and large deformation.^[^ ^11^, ^24^ ^]^ This instability adversely affects capacitance change and may result in an electrical breakdown, thereby diminishing the overall energy harvesting capacity and efficiency of the device. In contrast, the dual‐membrane structure incorporates two distinct DEC: a mechanical energy input unit (DEC_C_) and a mechanical energy output unit (DEC_A_). The dual‐membrane structure can effectively suppress EMI, while simultaneously enabling both energy harvesting and actuation capacities.

The operating principle involves a cyclic sequence of electromechanical transformations (Figure 1b,c). Initially, in the absence of a charge, the DEC_C_ undergoes cyclic deformation owing to external mechanical forces, leading to variations in its capacitance (*C*) from an initial capacitance (*C_Cinitial_*) to a maximum value (*C_Cmax_*). Consequently, an applied voltage (*V*) injects charges (*Q*) into the DEC_C_, generating electrostatic energy (*E*) during the stretching and releasing processes according to *E = Q^2^/2C*. As illustrated in Figure 1b, when released, the DEC_C_ increases in thickness, leading to a decrease in capacitance (*C*) and a subsequent increase in voltage according to the equation *Q* = *CV*. This phenomenon facilitates an increase in electrical energy, which can then be harvested through various discharge mechanisms. However, the process inherently increases the susceptibility to EMI owing to the occurrence of electrostatic stress exceeding the elastic stress of the DE membrane.^[^ ^18^ ^]^ This can result in localized thinning of the DE film, and ultimately can cause electrical breakdown during the release process.^[^ ^22^, ^23^, ^24^, ^35^ ^]^ Furthermore, the localized thinning impedes the complete relaxation of the DE film to its original, unstrained state (*C_Cinitial_*), limiting the achievable capacitance change. Consequently, EMI comprises both the breakdown voltage and restricted capacitance change, significantly impacting the overall energy harvesting capacity and efficiency.

In contrast to the single‐membrane structure, the dual‐membrane structure incorporates the DEC_A_ to facilitate charge sharing with the DEC_C_. As illustrated in Figure 1c, when the DEC_C_ reaches its maximum capacitance, both DECs reach electrical equilibrium. Upon release, the stored elastic energy within the DEC_C_ causes its capacitance (*C_C_*) to decrease and its voltage to increase, thereby establishing a potential gradient relative to the DEC_A_ (*C_A_*). This potential gradient drives a partial transfer of charge from the DEC_C_ to the DEC_A_, effectively diminishing the electrostatic force within the DEC_C_ while simultaneously generating an electrostatic force within the DEC_A_ to deform it. The capacitance changes in the two DECs exhibit opposing trends; that is, as *C_C_* decreases, *C_A_* increases. This synergistic capacitance behavior improves the DEC_A_’s charge‐sharing capacity, further promoting charge transfer and reducing the electrostatic force within the DEC_C_ during release. Meanwhile, the charge transfers from the DEC_C_ to the DEC_A,_ forming an electric current through a load. Conversely, when the DEC_C_ is stretched, the charge flows back from the DEC_A_ to the DEC_C_, causing the DEC_A_ to return to its initial state, accompanied by an increase in *C_C_* and a decrease in *C_A_*. Similarly, this can effectively diminish the electrostatic force within DEC_A_, whereas the simultaneous application of a constant strain can suppress EMI in the DEC_C_. Thus, this cyclical charge transfer and capacitance change enable continuous energy harvesting and actuation capabilities within the dual‐membrane structure. In addition, it allows the minimum capacitance (*C_Cmin_*) of the DEC_C_ to approach its initial capacitance (*C_Cinitial_*) more closely, remaining lower than the minimum capacitance (*C_Cmin_*) attainable in the single‐membrane structure at the same saturating voltage. Therefore, this charge‐transfer process can suppress the EMI in the two DECs and induce actuation behavior in the DEC_A_, thereby enabling mechanical energy output. Accordingly, based on the nonlinear field theory of elastic dielectrics, a thermodynamic model of the dual‐modal system was built to achieve the equilibrium equation between the elastic energy and the electrostatic energy (Note S1, Supporting Information).

Based on the above concepts, we constructed a dual‐modal DE system, consisting of a dual‐membrane structure, an electret electrostatic voltage source (EEVS), and a charge pump circuit, all interconnected electrically. As mentioned previously, the dual‐membrane structure consisted of two distinct DECs: a mechanical energy input unit (DEC_C_) and a mechanical energy output unit (DEC_A_), as depicted in Figure 1d. Two distinct DECs, DEC_C_ and DEC_A_, were fabricated using commercial 3 M very high bond (VHB) tape of 500‐µm thickness with a 3x radial pre‐stretch. Carbon grease served as a compliant electrode for both DECs, which were mounted on a circular acrylic frame for structural support and stability. The detailed fabrication process of the DECs is described in Figure S1 (Supporting Information) and the Experimental section. Serving as the mechanical energy input unit, the DEC_C_’s capacitance varied from 4.2 to 12.7 nF under 0.8 Hz mechanical stimulation (Figure S2, Supporting Information). The DEC_A_ had an initial capacitance of 2 nF. Photographs of the two DECs are shown in Figure S3 (Supporting Information). Apart from the dual‐membrane structure, the remaining components of the dual‐modal DE system, including the electret electrostatic voltage source (EEVS) and charge pump circuit, were identical to those used in the traditional single‐modal DE generator. The EEVS can confer positive and negative charges on two DEC complaint electrodes through a rectifier bridge owing to the electrostatic effect of the electret film (more detailed explanation in Figure S4 and Note S2, Supporting Information).^[^ ^36^, ^37^ ^]^ Based on the EEVS and the pump circuit, the system can pump charges and achieve a high voltage.^[^ ^20^, ^38^ ^]^ Additionally, two distinct commercial polypropylene film capacitors (48 and 24 nF, type: CBB) were integrated into the circuit, along with a Zener diode set at 4 kV for voltage stabilization. Upon exceeding the stability voltage, the Zener diode can discharge surplus charges, effectively preventing the dielectric breakdown of the elastomer film (more detailed explanation in Figure S5 and Note S2, Supporting Information). The default parameters were used unless otherwise mentioned. A schematic diagram of the voltage and current measurements is shown in Figure S6 (Supporting Information). A rectifier bridge, acting as the electrical energy output module, converts the alternating current to direct current for energy harvesting (Figures 1d and S7, Supporting Information).

Initially, the system operated as a single‐modal DE generator during the voltage‐boosting stage (Figure S8, Supporting Information). Upon reaching the voltage saturation stage, the DEC_A_, bearing a 200 g load, was electrically connected in parallel with the DEC_C_, thus establishing a dual‐modal DE system (Figure S9, Supporting Information). After the construction of a dual‐membrane structure, the system remained at a stable dynamic voltage from 2.33 to 4.0 kV with an output short peak current of ≈130 µA (Figures 1e and S10, Supporting Information). The DEC_A_ cyclically increased and decreased the load by 200 g at a height of 4 mm. To demonstrate the simultaneous energy harvesting and actuation capabilities, 120 LEDs were used while simultaneously handling a 200 g load (Figure 1f; Movie S1, Supporting Information). Notably, unlike previously reported generator‐powered actuator designs, the dual‐modal DE system achieved actuation without compromising the voltage or current levels.^[^ ^39^, ^40^ ^]^ Integration of short peak currents revealed that a transferred charge of 22.2 µC for the dual‐modal DE system, exceeding that of the single‐modal DE generator by 1.1 µC (Figure S10, Supporting Information). This indicates that the DEC_A_ does not act as a passive load but rather as an active component to improve the electric performance of the system.

Furthermore, to compare the breakdown voltage of the dual‐membrane and single‐membrane structures, the Zener diode as a voltage‐stabilizer was removed to test the breakdown voltage (Figure S11, Supporting Information). As the voltage increased, the traditional single‐modal DE generator experienced an electrical breakdown at ≈4.1 kV during the release phase (inset in Figure 1g), which is in agreement with previous reports.^[^ ^17^ ^]^ The dual‐modal DE system achieved a breakdown voltage of ≈4.5 kV, which exceeded that of the single‐modal DE generator by ≈400 V (Figure 1g). Meanwhile, for comparison, a maximum transferred charge of 33 µC can be obtained using the integrated current (Figure 1h), which is 32% higher than that of the single‐modal DE generator. As a result, the dual‐membrane structure can effectively suppress the EMI in the DEC_C_ while simultaneously enabling the DEC_A_ to generate mechanical energy output and improve the overall electric performance. The working principle is presented in more detail below.

### 2.2 Working Mechanism of the Dual‐Modal DE System

**Figure 2:**
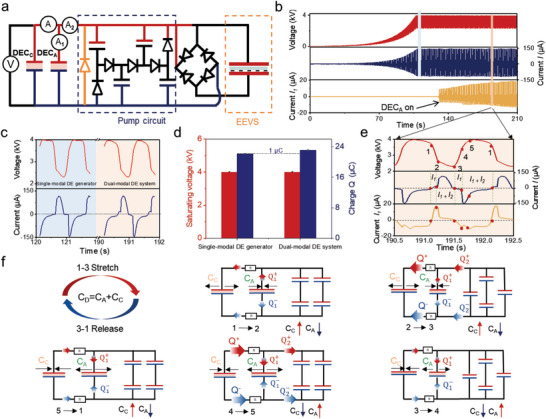
The working mechanism of the dual‐modal DE system. a) Test schematic of the voltage and electric current. Ammeter *A* and *A_1_* test the current in the DEC_C_ and in the DEC_A,_ respectively. b) Dynamic voltage and electric current under different structures. Single‐membrane structure converts to dual‐membrane structure after the DEC_A_ is turned on. c,d) Electric performance comparison of the single‐modal DE generator and dual‐modal DE system. e) Corresponding to the shaded areas of B. f) Schematic of the operating mechanism of the system. (Frequency: 0.8 Hz, the saturating voltage: 4 kV; DEC_C_’s capacitance varied from 4.2 to 12.7 nF).

To elucidate the working mechanism of the dual‐modal DE system, we compared the electric charge transfer behaviors of both the single‐modal DE generator and the dual‐modal DE system. As in the previous case, we incorporated the DEC_A_ into the single‐modal DE generator at the voltage saturation stage of 4 kV to fabricate the dual‐modal DE system (**Figures** **2**a and S9, Supporting Information). Throughout the testing phase, the electric current (labeled as “*I*”) signifies the inflow and outflow of electric charge within the DEC_C_, contributing to the system's electrical energy output. Furthermore, the second electric current (labeled as “*I_1_*”) represents the inflow and outflow of charge within the DEC_A_, prompting actuation behavior (Figure 2a–e). To delineate the intricate sequence of electromechanical transformations in the system, we graphically represented them on current‐time and voltage‐time plane (Figure 2f,g).

Upon activating the switch, the DEC_A_ began to work, forming a dual‐modal DE system (Figure 2a,b). Although the magnitudes of the voltage and current remained relatively constant, discernible changes were observed in their waveforms (Figures 2c and S12 and S13, Supporting Information). As mentioned above, the transferred charge reveals that the dual‐modal DE system is ≈1 µC larger than that of the single‐modal DE generator (Figure 2d). The specific procedure is explained in details below (Figure 2e,f). In the initial stage, the DEC_C_ and DEC_A_ operated at a high voltage (1). At this point, the DEC_C_ exhibited its minimum capacitance (*C_Cmin_*), whereas the DEC_A_ reached its maximum capacitance (*C_Amax_*) owing to the high voltage. Our approach involved a position‐controlled planar DEC_C_ unit subjected to mechanical stimuli, driven according to a cycle with the following two processes: stretch process (1‐3) and release process (3‐1) (Figure 2e,f).

Stretch process (1‐3): (1‐2) application of mechanical stress to the DEC_C_ caused an increase in its capacitance (*Cc*), resulting in a decrease in voltage. The voltage difference between the DEC_C_ and DEC_A_ causes a portion of the charges (*Q_1_*) stored in the DEC_A_ to flow into the DEC_C_, forming the current *I_1_* and resulting in an increase in the DEC_A_’s thickness and contraction of its area (Figure 2e,f). Consequently, the capacitance (*C_A_*) of the DEC_A_ decreased. Notably, in the single‐modal DE generator, no current was generated during this phase (Figures 2c and S12a, Supporting Information). (2‐3) As the voltage across the two DECs continued to decrease and fell below a specific threshold (2), a portion of the charges stored in the circuit were transferred to the DEC_C_, forming the current *I_2_*. Concurrently, the DEC_A_ continued to provide charge to the DEC_C_, thereby decreasing its area and increasing its thickness (Figure 2e,f). Therefore, the current *I* equals the sum of *I_1_* and *I_2_* (*I* = *I_1_+I_2_*). Eventually, external stimuli caused the DEC_C_ to expand and the DEC_A_ to contract, leading to State (3). At this point, *C_C_* reached its maximum value (*C_Cmax_*), whereas *C_A_* reached its minimum value (*C_Amin_*). Notably, the charges contributing to current *I* originated from both the circuit and the DEC_A_ during the stretch phase (1‐3) (Figure 2e).

Release process (3‐1): (3‐4) upon the removal of the applied mechanical stress, *C_C_* decreased, leading to an increase in voltage. The voltage difference between the DEC_C_ and DEC_A_ induced the flow of charges from the DEC_C_ to the DEC_A_, generating the current *I_1_*. The charge transfer process resulted in the expansion of the DEC_A_ area and a reduction in its thickness, consequently increasing *C_A_*. Similar to the stretching process, no current was generated in the single‐modal DE generator during this phase (Figures 2c and S12a, Supporting Information). (4‐5) As the voltage across the two DECs continued to increase and surpassed a certain threshold (4), the capacitors in the circuit switch to a parallel configuration. This configuration allowed a portion of the charges stored in the DEC_C_ to flow forward to the DEC_A_ (*I_1_*) and the circuit (*I_2_*), respectively, contributing to the total current *I* (*I* = *I_1_* + *I_2_*). (5‐1) With further contraction of DEC_C_, the voltage continued to increase, reaching a saturation point. In the single‐modal DE generator, the Zener diode maintained the voltage at this saturation level during this phase (Figures 2c and S13a, Supporting Information). However, high electrostatic stress under high voltage hindered the capacitance of the DEC_C_ from returning to its initial state (detailed in Note S3, Supporting Information). In contrast, in the dual‐modal DE system, the DEC_A_ shared a portion of the charge of the DECc, generating the current (*I_1_*) and causing a decrease in charge density during the contraction process (Figures 2e and S13b, Supporting Information). The cycle was completed upon reaching stage 1, with *C_C_* and *C_A_* at their minimum (*C_Cmin_*) and maximum capacitances (*C_Amax_*), respectively (Figure 2e). The transferred charge *Q_2_* (integrated by current *I_2_*) and the voltage change of the system can be calculated as follows (Note S3, Supporting Information):

(1) $$Q_{2}=\frac{C_{S\;}C_{Dmin}V_{H}\left(2n-3\right)}{nC_{Dmin}+3C_{S\;}}\;$$

(2) $$\frac{V_{5}}{V_{1}}=\frac{\left(2m^{2}+2.5\right)\left(3r+2nm^{2}+n\right)}{\left(2+2m^{2}\right)\left(n+8m^{2}+1\right)\left(2m^{2}+1\right)}\;$$

where *C_Dmax_* and *C_Dmin_* are the maximum and minimum capacitances of the DEC during the cycle, respectively; *n* and *V_H_* are the change ratio of *C_Dmax_*‐*C_Dmin_* and the voltage of the DEC, respectively; *C_S_* is the individual capacitor in the circuit; and *m* is the ratio of the *C_S_* and *C_Dmin_*. The capacitance of the dual‐membrane structure changed between *C_Dmax_* (*C_Amax_+C_Cmin_*) and *C_Dmin_* (*C_Cmax_* +*C_Amin_*) during mechanical cycling. Equations (1) and (2) demonstrate that the voltage variation and transferred charge (*Q_2_*) between the dual‐membrane structure and the circuit remain relatively stable within a specific range when the dual‐membrane structure exhibits a larger *C_Dmin_* and smaller *n* (Figure S14, Supporting Information). Furthermore, the charge redistribution (*Q_1_*) in the dual‐membrane structure enhanced the total transferred charge (*Q*) (Figure 2c,d; Figure S10, Supporting Information).

The charge transfer process in the dual‐modal DE system differed significantly from that in the single‐modal DE generator, particularly during phases (1‐2), (3‐4), and (5‐1) (Figure S12, Supporting Information). In the single‐modal DE generator, no inter‐DEC charge transfer occured during phases (1‐2) and (3‐4), because the DEC_C_’s charge remained constant the despite capacitance changes induced by the applied mechanical stress. Furthermore, during phase (5‐1), the single‐modal DE generator was discharged solely through the Zener diode. In contrast, the dual‐modal DE system exhibited dynamic charge sharing between the DEC_C_ and DEC_A_ throughout these phases. During phases (1‐2) and (3‐4), this charge sharing occured directly between the DEC_C_ and DEC_A_, whereas in phase (5‐1), the charge redistribution involved both the DEC_C_, DEC_A_, and Zener diode. Meanwhile, as mechanical stimuli, the opposite deformations of the two DECs resulted in opposite trends in their capacitance change (Movie S2, Supporting Information). Moreover, owing to its dual‐membrane structure, the dual‐modal DE system can decrease the charge concentration in the DEC_C_ during the release phase and improve the charge output (*Q*).

### 2.3 Effect of Structural Parameters on the Energy Harvesting and Actuation

**Figure 3:**
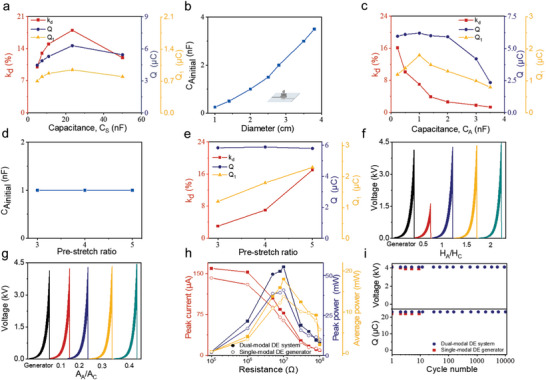
Relation between the energy harvesting and actuation. Actuation and electric performance includes the dynamic strain (*k_d_*) of the DEC_A_, the transferred charge (*Q*) of the system, and the charge variation (*Q_1_*) specific to the DEC_A_. a) *Q*, *Q_1_*, and *k_d_* as a function of individual capacitor *C_S_* in the circuit. b,c) *Q*, *Q_1_*, and *k_d_* as a function of the electrode diameter. b) Relationship between the initial capacitance of DEC_A_ (*C_Ainitial_*) and the electrode diameter. c) Dependence of *Q*, *Q_1_*, and *k_d_* on *C_Ainitial_*. d,e) *Q*, *Q_1_*, and *k_d_* as a function of the pre‐stretch ratio applied to DEC_A_. d) Relationship between *C_Ainitial_* and the pre‐stretch ratio. e) Influence of *C_Ainitial_* on *Q*, *Q_1_*, and *k_d_*. f,g) Breakdown voltage under varying different f) thickness ratio (*H_A_/H_C_*) and g) area ratio (*A_A_/A_C_*) between the DEC_C_ and the DEC_A_. h) Comparison of maximum electric performance achieved by the single‐modal DE generator and dual‐modal DE system. The saturation voltage of the single‐modal DE generator and dual‐modal DE system is 4.0 and 4.4 kV respectively. i) Continuous voltage and transferred charge test for the dual‐modal DE. system and the control single‐modal DE generator under the saturating voltage of 4.1 kV. (Frequency: 0.8 Hz; DEC_C_’s capacitance varied from 4.2 to 12.7 nF).

To further elucidate the relationship between actuation and energy harvesting performances, we investigated the interplay between the three key variables influencing both mechanisms (**Figure** **3**). We selected the transferred charge in the DEC_C_, denoted by *Q*, and the dynamic change in the area of the DEC_A_, denoted by *k_d_*, as the key parameters for evaluating the electric performance and actuation behavior, respectively.^[^ ^41^, ^42^ ^]^ *Q* can be obtained by integrating current *I*. *k_d_* can be calculated as follows:

(3) $$k_{d}=\frac{A_{1}-A_{3}}{A_{3}}\;\times 100\%\;$$

where *A_1_* is the electrode area of the DEC_A_ at one point during the cycle, and *A_3_* is the electrode area of the DEC_A_ at three points during a cycle. This can be assessed using a camera (Figure S15, Supporting Information). Moreover, to investigate the relationship between the transferred charge and dynamic change *k_d_*, we obtained the transferred charge *Q_1_* in DEC_A_ by integrating current *I_1_*. A schematic diagram of the test setup is shown in Figure 2a.

First, we investigated the effect of *Q* on both *Q_1_* and *k_d_* along with their relationship (Figure 3a). In order to make a consistent comparison, the DEC_A_ was fabricated using a 100‐µm thick VHB 9473 with a 4x radial pre‐stretch and an initial area of 0.78 cm^2^. The saturating voltage and mechanical frequency were set to 1.2 kV and 0.8 Hz, respectively. As the individual capacitor *C_S_* in the charge pump increases from 5 to 24 nF, the stored energy and *Q* in the system increase according to Equation (1) and Equations S1–S8 (Supporting Information). As the *Q* increased, so did the *Q_1_* and *k_d_*, which reached maximum values of 0.95 µC and 18%, respectively (Figures 3a and S16a, Supporting Information). When the individual capacitor *C_S_* exceeds 24 nF, the performance decreased. This can be attributed to significant charge leakage in the larger individual capacitors, which cannot be ignored. In other words, the DEC_A_ shared more charge as *Q* increased in the DEC_C_. Meanwhile, the increased charge transferred to the DEC_A_ led to a greater electrostatic force, resulting in larger deformation of *k_d_* and a more substantial capacitance change in the DEC_A_ (Figure 3a; Note S4 and Figure S16b, Supporting Information).

We further employed two distinct methods to modify the structural parameters of DEC_A_ to investigate the interplay between the two DECs (Figure 3b–e). The first approach involves adjusting the diameter of DEC_A_ to alter its capacitance. As the diameter increases from 1 to 3.8 cm, the initial capacitance of DEC_A_ increases from 1 to 3.5 nF (Figure 3b). However, the three parameters exhibited different trends. As the DEC_A_’s capacitance (*C_A_*) increased from 0.3 to 2 nF, *Q* and the saturating voltage remained relatively constant. However, when capacitance *C_A_* of the DEC_A_ reached 3 nF, the performance diminished (Figures 3c and S17a, Supporting Information). This decline is attributed to insufficient capacitance change within the dual‐membrane structure, falling below the critical stability threshold of a 1.5 capacitance change ratio, consequently compromising the ability of the dual‐modal DE system to maintain charge pump functionality.^[^ ^43^ ^]^ Furthermore, as the diameter increased, the dynamic area strain *k_d_* decreased owing to the large initial area. However, the area changes of the DEC_A_ initially increased and then decreased, resulting in a corresponding trend in the capacitance change and charge transfer to the DEC_A_ (Figure S17b, Supporting Information). At *C_A_* = 2 nF, *Q_1_* reached a maximum value of 1.8 µC, which shared 29% of the *Q*. However, as the initial radius of the DEC_A_ surpassed 2 cm (*C_Ainitial_>*1 nF), the resulting larger initial area led to a decrease in both *Q_1_* and capacitance variation (Figure S17b, Supporting Information). This observation can be attributed to the direct coupling between charge density and deformation, as well as the operating regimen of the dual‐membrane structure, where the stored electrical energy becomes comparable to the mechanical strain energy. The other approach is to reduce the thickness of the DEC_A_ by increasing its pre‐stretch ratio while maintaining a constant initial capacitance of 1 nF (Figures 3d,e and S18, Supporting Information). Meanwhile, owing to the elimination of the pull‐in instability by the transverse prestress, the DEC_A_ was more easily deformable, improving the capacitance variation of the DEC_A_ and providing better charge sharing (Figure S18, Supporting Information).^[^ ^21^ ^]^ Therefore, although *Q* remained constant, *Q_1_* and *k_d_* exhibited significant improvements with increasing pre‐stretch ratios (Figure 3e).

Furthermore, we investigated the actuation and electric performances under different humidity levels and mechanical frequencies (Figures S19–S22, Supporting Information). Because of the protection of the carbon grease electrode, the electrical and actuation performances were stable under different humidity levels (Figures S19 and S20, Supporting Information).^[^ ^21^ ^]^ However, as the frequency changed, the electrical and actuation performances decreased slightly, owing to the viscous hysteresis of the dielectric elastomer membrane (Figures S21 and S22, Supporting Information).^[^ ^21^ ^]^ Moreover, we studied the influence of the saturation voltage on the performance of the dual‐modal DE system (Figures S23 and S24, Supporting Information). *Q* increased with an increase in the saturation voltage, which is consistent with Equation (1). This implies a higher quantity of electric charge and a larger charge transfer in the DECs. Thus, the DEC_A_ can share more charges, and both *Q_1_* and *k_d_* increase (Figure S23, Supporting Information). Eventually, when the saturation voltage reached 1.6 kV, the maximum values of *Q* and *k_d_* were 6.9 µC and 27%, respectively. However, the achievable voltage was limited by the low breakdown strength of the thin DEC_A_ films. Based on the above results, we employed two distinct approaches‐utilizing the thickness ratio (*H_A_/H_C_*) and area ratio (*A_A_/A_C_*) of the DEC_A_‐DEC_C_ couple to suppress EMI and improve the breakdown voltage of the dual‐modal DE system (Figure 3f,g). When *H_A_/H_C_* = 1 and *H_A_/H_C_* > 1, the breakdown voltage of the system was higher than that of the single‐modal DE generator with a single‐membrane structure (Figure 3f). At *H_A_/H_C_* = 1, the two DECs shared charges with each other and suppressed their respective EMI, resulting in an improvement in the breakdown voltage of the dual‐modal DE system. At *H_A_/H_C_* > 1, an increase in the thickness of DEC_A_ caused it to bear a larger charge to obtain high breakdown voltage. However, when *H_A_/H_C_* < 1, the breakdown voltage of the dual‐modal DE system was lower than that of the single‐modal DE generator, which was attributed to the high electric field in the DEC_A_ (Figure S25, Supporting Information). Moreover, increasing the area of the DEC_A_ enhanced its capacitance, enabling it to share a greater portion of the charge from DEC_C_. This effectively suppressed the EMI within the DEC_C_ and, consequently, improved the breakdown voltage of the dual‐modal DE system (Figure 3g). Eventually, a high breakdown voltage of 4.5 kV was obtained, surpassing that of a traditional single‐modal DE generator with a single‐membrane structure of over 0.4 kV. These results were consistent with those of our previous simulation study (Figure S26, Supporting Information).

Based on the above principles, we improved the initial capacitance ratio of the DEC_A_ to 0.85 nF (*H_A_/H_C_* = 2 and *A_A_/A_C_* = 0.4). Other than actuation behavior, as a comparison with the single‐modal DE generator, the dual‐modal DE system increased electric performance at 0.8 Hz by a factor of over 30% (Figure 3h; Note S4, Supporting Information, transferred charge, peak power, and average power increased from 23.5 µC, 40.90 mW, and 13.84 mW to 31 µC, 55.44 mW, and 18.01 mW, respectively). Finally, we performed fatigue measurements on the single‐modal DE generator and dual‐modal DE system under a saturation voltage of 4.1 kV (Figures 3i and S27, Supporting Information). The dual‐modal DE system retained up to ≈95% of its initial charge transfer after >10 000 cycles, whereas the control single‐modal DE generator suffered a breakdown after only ≈10 cycles. Silicone elastomers constitute an additional class of extensively utilized DEs that do not undergo pre‐stretching treatment because of their inherent high extensibility and low tackiness. Notably, the results obtained from these materials are consistent with those derived from pre‐stretched acrylic elastomers. A silicone‐based DE system with a dual‐modal configuration demonstrated comparable performance enhancements, exceeding that of the silicone‐based single‐modal DE generator by over 40% (Figure S28 and Note S4, Supporting Information, transferred charge, peak power, and average power increased from 6.27 µC, 24.20 mW, and 7.01 mW to 8.80 µC, 33.88 mW, and 9.80 mW, respectively).

### 2.4 Application Demonstrations for the Dual‐Modal DE System

**Figure 4:**
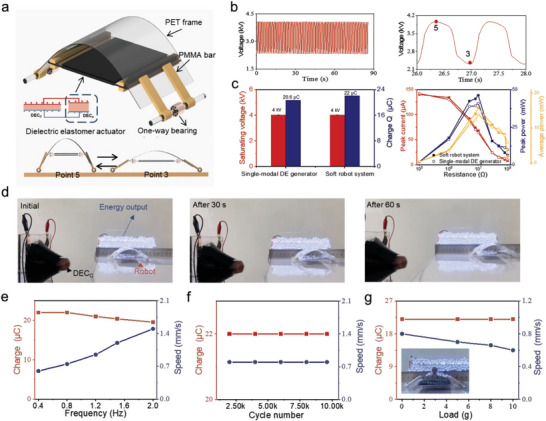
Characterization of the soft robot system. (a) Design of the soft robot with a uni‐directional DEC_A_. a,b) Schematic of the robot with the DEC_A_, and phases of the actuation at two different voltages point (V_3_ < V_5_). c) Comparison of output electric performance achieved by the soft robot system and the control single‐modal DE generator. d) Optical images of the side view of the operation during an actuation cycle. e–g) Speed of the soft robot and transferred charge of the system under various conditions. (e) Different frequency. (f) Different cycle numbers. (g) Different loads.

The exceptional flexibility and lightweight nature of dielectric elastomers render them suitable for soft robotics applications. Furthermore, this system can supply electric energy and actuation power, facilitating robot patrolling and detection in remote and hard‐to‐access locations. Here, we demonstrated that the DEC_A_ serves as an actuator that enables the motion of a soft crawl robot (**Figure** **4**a–d). The initial capacitance of the DEC_A_ was 1.5 nF. Alongside the DEC_A_, the robot included a flexible arc‐shaped body and two unidirectional bearing wheels to facilitate directional motion (Figure 4a). The resulting soft robot had dimensions of 50 mm × 60 mm × 40 mm and a weight of 10.8 grams (Figure S29, Supporting Information). Detailed information regarding the specific preparation process of the robot can be found in Figure S30 (Supporting Information). In its initial state, owing to the constraints imposed by the flexible arc‐shaped frame, the DEC_A_ maintained a pre‐stretched state and uni‐directional in‐plane strain in the actuation direction. As the mechanical stimuli were applied/removed to/from the DEC_C_, the resulting voltage changes caused the DEC_A_ to contract/elongate (Figure 4a,b). This, in turn, led to the dynamic bending of the arced frame, resulting in a quasi‐linear displacement. Owing to the one‐way bearings in the wheels, the forward displacement was sustained throughout the contraction and elongation cycles of the DEC_A_, ultimately generating the forward crawling motion of the soft robot (more detail in Note S5 and Figure S31, Supporting Information). Meanwhile, the charge entered and left the DEC_C_ to form an alternating electric current (*I*). More importantly, whilst maintaining actuation behavior, the transferred charge, peak power, and average power of the system reached 22 µC, 46.2 mW, and 15.8 mW, which were over 5% above that of the single‐modal DE generator (Figure 4c). Dual‐modal DE system demonstrated the ability for the robot to crawl at 0.85 mm s^−1^ while simultaneously illuminating 120 LEDs (Figure 4d; Movie S3, Supporting Information). Under continuous mechanical stimulation, the robot covered a distance of 7 cm within 80 s. Furthermore, as the frequency of the mechanical stimuli applied to the DEC_C_ increased, the crawling speed also increased, whereas the transferred charge experienced a slight decrease (Figure 4e; Movie S4, Supporting Information). The decrease in the transferred charge was attributed to the viscous hysteresis inherent to the DE membrane. Notably, both the crawling speed and the transferred charge remained stable during the extended cyclic operation (Figure 4f). Additionally, the device demonstrated the ability to carry external loads while maintaining electrical energy output (Figure 4g). However, owing to the presence of an external load, the crawling speed decreased compared with the unloaded condition. Specifically, under increasing external loads ranging from 0 to 10 g, the robot's crawling speed decreased from 0.8 to 0.6 mm s^−1^, while the transferred charge remained constant at 22 µC (Figure 4g; Movie S5, Supporting Information). This decrease in speed was attributed to the increased unidirectional in‐plane strain experienced by the DEC_A_ when subjected to higher external loads.

**Figure 5:**
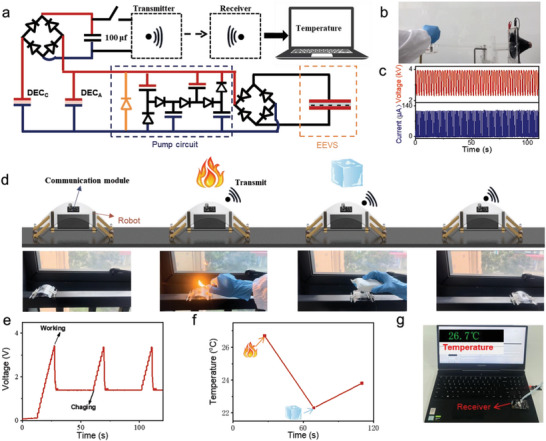
Illustration of the mechanically powered patrol robot system. a) Schematic diagram of the mechanically powered patrol robot system. b) Photo images of hand‐shaking powered generation device. c) Output voltage and electric current of the hand‐shaking power generation device under the saturating voltage of 4 kV. d) Soft robot equipped with temperature sensors crawls through different locations and transmits real‐time temperature information. e–g) Receiver node of mechanically powered patrol robot system. (e) Voltage variation of the capacitor (100 µF) in consequent temperature reading processes. (f) Temperature changes at different locations. (g) A computer interface showing the successful transmission of the temperature information.

To demonstrate the practical applications of the dual‐modal DE system, we integrated it into a crawling robot equipped with a wireless temperature reading module for patrolling and environmental monitoring in complex settings (**Figure** **5**a). The parameters of the dual‐modal DE system and crawling robot were consistent with those described above. The crawling robot served as a patrol unit to monitor the complex environment, whereas the hand‐rotating shaft provided continuous mechanical stimulation to the DEC_C_ at a frequency of 1 Hz (Figure 5b). The resulting voltage and current outputs from this hand‐shaking power generation system are shown in Figure 5c. A 100 µF capacitor, connected to the system through a rectifier, stored the harvested electrical energy from the mechanical stress, acting as a power source for the wireless temperature reading module. Upon continuous application of the 1 Hz mechanical stimulus to the DEC_C_, the robot, equipped with the wireless temperature reading module, moved forward (Figure 5d; Movie S6, Supporting Information). Simultaneously, the capacitor was charged from 0 to 3.6 V within ≈15 s, providing sufficient power for the wireless transmitter to emit a temperature signal (Figures 5e and S32, Supporting Information). The robot, equipped with a wireless transmitter, crawled on a narrow railing and, in turn, passed through flames and ice to eventually reach the designated location. Throughout its journey, real‐time temperature data were transmitted from the three distinct environments. Variations in the transmitted ambient temperature are shown in Figure 5f.

## 3 Conclusion

This study demonstrates a dual‐membrane strategy to overcome the limitations of EMI in DE systems and achieve simultaneous energy harvesting and actuation. By leveraging the charge‐sharing mechanism of the dual‐membrane structure, the dual‐modal DE system effectively transfers the electrostatic force between the two membranes, mitigating the charge concentration during the release process while simultaneously enabling actuation and enhancing the breakdown voltage. This results in a significant improvement in the electrical output performance. Investigation of the charge transfer behavior revealed the crucial interplay between the actuation performance, transferred charge within the system, and transferred charge within the dual‐membrane structure. Notably, the system can generate both electrical and mechanical energy without compromising performance; therefore, it opens doors for diverse applications, particularly in generators and soft robotics. For instance, by harnessing ambient vibrations from human activity, the system can provide a continuous supply of mechanical energy, thereby enabling the self‐powered operation of soft robots. This self‐sufficiency eliminates the reliance on external power sources and batteries, making the system particularly well suited for deployment in remote or hard‐to‐access locations where battery replacement or recharging is impractical. Future research can explore the further optimization of material properties, structural designs, and control strategies to enhance the efficiency, durability, and adaptability of dual‐modal DE systems.

## Conflict of Interest

The authors declare no conflict of interest.

## Author Contributions

Z.Z. and W.H. contributed equally to this work. Z. X. and S.E. designed the research. Z.Z. and W.H. carried out the fabrication, characterization, and data analysis. Z.X. and Z.Z. wrote the manuscript. S.E. guided the project. S.Z., J.T., J.C., and J.C. helped with the experiments. All authors reviewed and commented on the manuscript.

## Supporting information



Supporting Information

Supplemental Movie 1

Supplemental Movie 2

Supplemental Movie 3

Supplemental Movie 4

Supplemental Movie 5

Supplemental Movie 6

## Data Availability

The data that support the findings of this study are available from the corresponding author upon reasonable request.
