# Supplementary material for: Dual‐Modal Dielectric Elastomer System for Simultaneous Energy Harvesting and Actuation
Source: Adv Sci (Weinh). 2024 Dec 11;12(5):2410724. doi: 10.1002/advs.202410724 (PMC11791972; doi:10.1002/advs.202410724)
Supplement: Supplementary file 1 — Supporting Information [file ADVS-12-2410724-s006.docx]

**Supporting Information**

Dual-Modal Dielectric Elastomer System for Simultaneous Energy Harvesting and Actuation

Zhiyuan Zhang^1,2†^, Wenwei Huang^1,2†^, Shaodi Zheng^1,2^, Jianbo Tan^1,2^, Jinzhan Cheng^1,2^, Jiancheng Cai^1,2^, Shiju E^1,2*^, Zisheng Xu^1,2*^

*1 Key Laboratory of Urban Rail Transit Intelligent Operation and Maintenance Technology & Equipment of Zhejiang Province, College of Engineering, Zhejiang Normal University, Jinhua 321004, People’s Republic of China*

*2 Jinhua Intelligent Manufacturing Research Institute, Jinhua 321004,* *People’s Republic of China*

^†^ *These authors contributed equally to this work*

*e-mails: [zishengxu@zjnu.edu.cn](mailto:zishengxu@zjnu.edu.cn); [shiju.e@zjnu.edu.cn](mailto:shiju.e@zjnu.edu.cn)

**Supplementary Note 1：**

In the reference state, the capacitor is uncharged and undeformed with an initial area *A* and thickness *H*. In the actuated state, the capacitor is charged with a voltage $\Phi$ and charge *Q*, and the capacitor area is expanded from *A* to *a*, meanwhile the thickness is decreased from *H* to *h*. The deformation is equal-biaxial with a stretch$\lambda=\sqrt{a/A}$. For such a thin-membrane capacitor, the electric field (*E*) and electric displacement (*D*) are $\Phi/h$ and $Q/a$, respectively. In this case, the self-charged capacitor system can be considered as a closed thermodynamic system.^[1.2]^ Thus, the Helmholtz free energy density of this system can be assumed by:

$W=\frac{\mu}{2}(2\lambda^{2}+\lambda^{-4}-3)+\frac{D^{2}}{2\varepsilon}$ (1-1)

where $\mu$ is the shear modulus and $\varepsilon$ is (absolute) permittivity. And the free energy includes: 1) Elastic energy described by the neo-Hookean model and 2) Electrostatic energy described by the linear dielectric model, $D=\varepsilon E$. Furthermore, the dielectric elastomer is taken to be incompressible, that is, $HA=ha$, so that, $h=H\lambda^{-2}$. Thus, the free energy of the single-membrane structure can be expressed by:

$F_{Q}=HAW=\frac{\mu HA}{2}(2\lambda^{2}+\lambda^{-4}-3)+\frac{Q^{2}H}{2\varepsilon A}\lambda^{-4}$ (1-2)

The free energy of the single-modal system is the sum of the free energy of the single-membrane structure and the free energy of the charge pump circuit, which can be expressed by:

$F=\frac{\mu HA}{2}\left( 2\lambda^{2}+\lambda^{-4}-3 \right)+\frac{Q^{2}H}{2\varepsilon A}\lambda^{-4}+ \frac{4}{3}C_{S}V^{2}$ (1-3)

Where $C_{S}$ is the individual capacitor in the circuit, $V$ is the voltage of capacitor $C_{S}$.

At a fixed charge, the free energy of the single-membrane structure as a function of the stretch, $F_{Q}(\lambda$*）*. Assume $dF_{Q}/d\lambda=0$ with $Q$ held constant. The equation of state is given by:

$Q^{2}=\mu\varepsilon A^{2}(\lambda^{6}-1)$ (1-4)

For charge-controlled actuation, charge is gradually added to the homogeneous system, a single stable state changes continuously, and no instability occurs. The maximum actuation stretch is limited by electrical breakdown field. By setting $dF_{Q}/d\lambda=0$ in (1-2), the critical stretch $\lambda_{c}=2^{1/3}$, critical charge $Q_{c}=A\sqrt{3\mu\varepsilon}$, and critical electric field $E_{c}=2^{{-2}/3}\sqrt{3\mu/\varepsilon}$ are obtained. A membrane of neo-Hookean material will become thinner, resulting in electrical breakdown.

The dual-membrane structure can be simplified by representing it with two individual capacitors connected in parallel (DEC_C_ and DEC_A_). Similarly, the free energy of the dual-membrane structure is a sum of the free energies of both capacitors, namely

$F=\frac{\mu H_{C}A_{C}}{2}(2\lambda_{C}^{2}+\lambda_{C}^{-4}-3)+\frac{{Q_{C}}^{2}H_{C}}{2\varepsilon A_{C}}{\lambda_{C}}^{-4}+\frac{\mu H_{A}A_{A}}{2}(2\lambda_{A}^{2}+\lambda_{A}^{-4}-3)+\frac{{Q_{A}}^{2}H_{A}}{2\varepsilon A_{A}}{\lambda_{A}}^{-4}$ (1-5)

where $A_{C}$ and $H_{c}$ are the initial area and thickness of DEC_C_, $A_{A}$ and $H_{A}$ are the initial area and thickness of DEC_A_, respectively. $\Phi$ is the voltage of DEC_C_ and DEC_A_ in the actuated state. $Q_{C}$, $Q_{A}$, $\lambda_{C}=\sqrt{a_{c}/A_{C}}$ and $\lambda_{A}=\sqrt{a_{A}/A_{A}}$ are the charge and stretch of DEC_C_ and DEC_A_ in the actuated state, respectively.

In this case, the two capacitors have the same thickness *H*, and the effect of boundary in between DEC_C_ and DEC_A_ is neglected. The charges on the two capacitors are $Q_{A}=a_{A}D_{A}=\varepsilon{\lambda_{A}}^{4}A_{A}\Phi/H$ and $Q_{C}=a_{C}D_{C}=\varepsilon{\lambda_{C}}^{4}A_{C}\Phi/H$. Once the capacitor receives electric charge, the power source is disconnected, so that the total charge $Q$ on the DEC_C_ and DEC_A_ is fixed, namely

$Q=Q_{A}+Q_{C}=\varepsilon({\lambda_{A}}^{4}A_{A}+{\lambda_{C}}^{4}A_{C})\Phi/H$ (1-6)

As to the two parallel capacitors, the coefficient of the charge-voltage relation (1-6) is their combined capacitance, $C=\varepsilon({\lambda_{A}}^{4}A_{A}+{\lambda_{C}}^{4}A_{C})/H$. Thus, the free energy of the dual-membrane structure can be expressd as:

$F_{Q}=\frac{\mu HA_{A}}{2}(2\lambda_{A}^{2}+\lambda_{A}^{-4}-3)+\frac{\mu HA_{C}}{2}(2\lambda_{C}^{2}+\lambda_{C}^{-4}-3)+\frac{{HQ}^{2}}{2\varepsilon{{(\lambda}_{A}}^{4}A_{A}+{\lambda_{C}}^{4}A_{C})}$ (1-7)

Based structural parameters of charge pump circuit during the releasing process, the free energy of the dual-modal DE system can be expressed by:

$F=\frac{\mu HA_{A}}{2}(2\lambda_{A}^{2}+\lambda_{A}^{-4}-3)+\frac{\mu HA_{C}}{2}(2\lambda_{C}^{2}+\lambda_{C}^{-4}-3)+\frac{{HQ}^{2}}{2\varepsilon{{(\lambda}_{A}}^{4}A_{A}+{\lambda_{C}}^{4}A_{C})}+\frac{4}{3}C_{S}V^{2}$ (1-8)

Setting $\partial F_{Q}(\lambda_{A},\lambda_{C})/{\partial\lambda}_{A}=0$ and $\partial F_{Q}(\lambda_{A},\lambda_{C})/{\partial\lambda}_{C}=0$ with the total charge $Q$ held constant, we obtain two equations of state:

${\varepsilon\Phi}^{2}=\mu H^{2}({\lambda_{A}}^{-2}-{\lambda_{A}}^{-8})$ (1-9)

${\varepsilon\Phi}^{2}=\mu H^{2}({\lambda_{C}}^{-2}-{\lambda_{C}}^{-8})$ (1-10)

As the total charge $Q$ increases, the dual-modal system undergoes a series of equilibrium states governed by Eq. (1-9) and (1-10), $\lambda_{A}=\lambda_{C}$ is a trivial solution of Eq. (1-9) and (1-10), representing the homogeneous deformation of the dual-modal system. Solutions also can be obtained for two branches of inhomogeneous deformation with $\lambda_{A}<\lambda_{C}$ and $\lambda_{A}>\lambda_{C}$. By calculating the free energy of the composite system, we find that the solutions for inhomogeneous deformation are always more stable than for the homogeneous deformation. For the dual-modal system, the critical condition of stability is

$\left| \begin{matrix} \frac{\partial^{2}F_{Q}}{\partial{\lambda_{A}}^{2}} & \frac{\partial^{2}F_{Q}}{\partial\lambda_{A}\lambda_{C}} \\ \frac{\partial^{2}F_{Q}}{\partial\lambda_{A}\lambda_{C}} & \frac{\partial^{2}F_{Q}}{\partial{\lambda_{C}}^{2}} \end{matrix} \right|$*=0*  (1-11)

**Supplementary Note 2:**

**Electret electrostatic voltage source (EEVS):**

An electret electrostatic voltage source (EEVS), which is composed of a charged PTFE electret film and two electrodes, is used as an initial charge source. The PTFE film (thickness of 50 µm) was charged under air conditions with the assistance of a metal grid and 8 cm away from the PTFE film through negative corona polarization treatment (**Figure S4a, Supporting Information**). The PTFE film kept a net negative electrostatic charge and stabilized at approximately the surface potential of the -0.55 kV (**Figure S4b, Supporting Information**). Assuming the electrostatic charges ($\sigma$) on the surface of the PTFE and electrodes are uniformly distributed, at any equilibrium state, the surface charge density$\sigma_{2}$ and $\sigma_{2}$ on the upper electrode and the bottom electrode are given as follows,^[^[^3^](#_ENREF_30)^]^ respectively (**Figure S4c, Supporting Information**):

$\sigma_{2}=\frac{\sigma d_{1}}{d_{2}\varepsilon_{r}+d_{1}}$ (2-1)

$\sigma_{1}=\sigma{-\sigma}_{2}$ (2-2)

where *d_1_* is the thickness of the PTFE; $\varepsilon_{r}$ is the relative permittivity of the PTFE; $d_{2}$ is the distance between the PTFE surface and the bottom electrode. Due to the charge difference, a voltage can be built between two electrodes. When the DEC is connected to the EEVS via a rectifier (**Figure S4c, Supporting Information**), an initial voltage of around 11 V is observed in the DEC through a voltmeter (**Figure S4d, Supporting Information**). Once mchanical stress is applied on the DEC, the inceasing capacitance leads to the voltage to drop to approximately 7 V (**Figure S4d, Supporting Information**). As cyclic mechanical stretch, the voltage dynamically varied between 7 and 11 V. When the DEC is in parrallel with the charge pump circuit, the DE system can self-pump charge and self-prime voltage.

**Mechanism of the Zener diode:**

The Zener diode is designed to operate in reverse breakdown mode. This means that when the voltage across the diode exceeds a specific threshold, known as the Zener voltage (*Vz*), the diode starts conducting and allows current to flow. Therefore, the Zener diode is connected in parallel with the DECs in the circuit. This configuration ensures that when the voltage across the DECs surpasses *Vz*, the excess charge carriers (electrons or holes) are diverted from the DECs to the Zener diode. The excess charge carriers accumulate in the depletion region of the Zener diode and undergo recombination, effectively neutralizing them. Once the voltage across the DECs falls below *Vz*, the charge dissipation ceases, and the Zener diode returns to its non-conducting state. This charge release process prevents further voltage increase across the DECs during the releasing process, maintaining it below the Zener voltage and protecting the DE film from electrical breakdown.

**Supplementary Note 3:**

The capacitance, voltage, and charge at different states can be denoted as follows: DE system: $C^{x}$, $V^{x}$, and $Q^{x}$; DEC_C_:$C_{Dx}$, $V_{Dx}$, and $Q_{Dx}$; charge pump circuit: $C_{Sx}$, $V_{Sx}$, and $Q_{Sx}$. The subscript *x* is used to denote the point index. For the charge pump circuit, $\frac{C_{S}}{C_{M}}=0.5$, the capacitance values in series and in parallel are given by $C_{ss}=\frac{2C_{S}C_{M}}{C_{S}+C_{M}}$ and $C_{sp}=2C_{S}+\frac{C_{M}}{2}$, respectively. The leakage losses are neglected to simplify the derivation in this work.

The voltage-boost process can be divided into four sub-stages: 1-2, 2-3, 3-4, and 4-5, as is shown in **Figure S8** (**Supporting Information**). The capacitance and voltage of DEC_C_ appears as *C_Dmin_* and *V_D1_* at point 1.

1-2: When the strain is applied on the DEC_C_, the capacitor (*C_c_*) of the DEC_C_ rises while the voltage (*V_D_*) of the DEC_C_ decreases, inducing a voltage difference between the DEC_C_ and the charge pump circuit in series. The electrical performance of the single-modal generator can be expressed according to the following equations.

At point 1, the voltages of DEC_C_ and the charge pump circuit are equal:

$V_{D1}=V_{s1}$ (3-1)

At unstretched state, the capacitance of DEC_C_ is at the minimum level $C_{D_{min}}$:

$C_{D1}=C_{D_{min}}$ (3-2)

The charge $Q_{D1}$in the DEC_C_ is

$Q_{D1}=C_{D_{1}}V_{D1}=C_{D_{min}}V_{D1}$ (3-3)

Due to Equation (3-1) ($V_{D1}=V_{s1})$, the charge $Q_{S1}$in the charge pump circuit is

$Q_{S1}=C_{SS}V_{S1}=C_{SS}V_{D1}=\frac{2C_{M}C_{S}}{C_{M}+C_{S}}V_{D1}$ (3-4)

According to Equation (3-2)-(3-4), at point 1, the total charge $Q^{1}$in the single-modal generator can be described:

$Q^{1}={Q_{D1}+Q_{s1}=C}_{Dmin}V_{D1}+C_{SS}V_{D1}=C_{D_{min}}V_{D1}+\frac{2{V_{D1}C}_{S}C_{M}}{C_{S}+C_{M}}$ (3-5)

During the process from point 1 to point 2, charges can not flow through two loads from the charge pump circuit to the DEC_C_ for the reverse bias of the diode *D_1_* and *D_3_*. Thus, the total charge is constant. Until the voltage of the DEC_C_ is lower than the voltage of any individual capacitor in the charge pump circuit, which causes the diodes *D_2_*, *D_4_* and *D_5_* to conduct, the charge pump circuit switches from series configuration to parallel configuration. Therefore, the point 2 is a critical point, as shown in **Figure S8b** (**Supporting Information**). The voltages of DEC_C_ and the charge pump circuit are equal and can be expressed as follows:

$V_{D2}=V_{s2}=V_{cs}=\frac{C_{M}}{C_{M}+C_{S}}V_{D1}$ (3-6)

During the process from point 1 to point 2, no charge flows between the charge pump circuit and DEC_C_. Therefore, the charge $Q_{D2}$in the DEC_C_ is equal to $Q_{D1}$:

$Q_{D2}=Q_{D1}=C_{D_{min}}V_{D1}$ (3-7)

However, due to the switching the charge pump circuit from series configuration to parallel configuration, the charge $Q_{S2}$ of the charge pump circuit is

$Q_{S2}=(2C_{s}+{\frac{1}{2}C}_{M})\frac{C_{M}}{C_{M}+C_{S}}V_{D1}=\frac{3C_{S}C_{M}}{C_{M}+C_{S}}V_{D1}$ (3-8)

According to Equation (3-8)-(3-9), the total charge $Q^{2}$in the single-modal generator can be described:

$Q^{2}={Q_{D2}+Q_{S2}=C}_{Dmin}V_{D1}+\frac{3C_{M}C_{S}}{C_{M}+C_{S}}V_{D1}$ (3-9)

According to Equation (3-5) and (3-9), after the switching of charge pump circuit configuration, the charge increased $\Delta Q^{1-2}$ can be expressed:

$\Delta Q^{1-2}=Q^{2}-Q^{1}=\frac{C_{M}C_{S}}{C_{M}+C_{S}}V_{D1}$ (3-10)

2-3: The voltage of the DEC_C_ continues to decrease with the stretching of DEC_C_. Charges flow through two loads from the charge pump circuit to the DEC_C_ (**Figure S8, Supporting Information**). Until the stretch ceased at point 3, the capacitance of DEC_C_ and the charge pump circuit appear as the maximum *C_Dmax_* and parallel configuration$C_{sp}=2C_{S}+\frac{C_{M}}{2}$, respectively, and the voltages of the DEC_C_ and the charge pump circuit are equal.

At point 3, the charge $Q_{D3}$ and the voltage $V_{D3}$ of the DEC_C_ is

$Q_{D3}=Q_{D2}+Q_{T}=C_{Dmin}V_{D1}+Q_{T}$ (3-11)

$V_{D3}=\frac{C_{Dmin}V_{D1}+Q_{T}}{C_{Dmax}}$ (3-12)

Due to $Q^{2}=Q^{3}$ and $V_{D3}=V^{3}$, according to Equation (3-9)-(3-12), the shuttling charge$Q_{T}$ between the DEC_C_ and the charge pump circuit can be expressed:

$Q_{T}=\frac{{(C}_{M}+C_{S}{)C}_{Dmin}C_{Dmax}+3{C_{Dmax}C}_{M}C_{S}}{{(C}_{M}+C_{S})(C_{Dmax}+2C_{S}+\frac{C_{M}}{2})}V_{D1}-C_{Dmin}V_{D1}$ (3-13)

The capacitance change ratio $n$ ($n=\frac{C_{Dmax}}{C_{Dmin}}$) of DEC_C_ is commonly used to express the ratio of the maximum capacitance of DEC_C_ $(C_{Dmax})$ and the unstretched capacitance $(C_{Dmin})$:

$Q_{T}=C_{Dmin}V_{D1}(\frac{C_{Dmin}}{C_{Dmin}+\frac{{2C}_{s}}{n}+\frac{C_{M}}{2n}}+\frac{3C_{M}C_{S}}{{(C}_{M}+C_{S})(C_{Dmin}+\frac{{2C}_{s}}{n}+\frac{C_{M}}{2n})}-1)$ (3-14)

3-4: Conversely, when the DEC_C_ is released, the capacitance of the DEC_C_ decreases while the voltage of DEC_C_ increases. Inducing a voltage difference between the DEC_C_ and the charge pump circuit in parallel. As the reverse bias of diodes *D_1_*and *D_3_*, there is no charge flow from the DEC_C_ to charge pump circuit until the voltage of DEC_C_ is higher than the voltage of two capacitors in series. It will cause the diode *D_1_* and *D_3_* to conduct, the parallel configuration charge pump circuit switches into series configuration. Thus the point 4 is an another critical point of the configuration transition as shown in **Figure S8d** (**Supporting Information**). Meanwhile, conservation of charge requires that charges on two plates of the individual capacitors keep equal magnitude. Thus, the positive and negative charge carriers in the diode *D_2_* separate from each other, compensating for charges of two adjacent plates of two capacitors, respectively. During the process from point 3 to point 4, there are no charge flows between the charge pump circuit and DEC_C_, and the charge $Q_{D4}$in the DEC_C_ is equal to $Q_{D3}$.

Due to $Q_{D4}=Q_{D3}$, the voltage $V_{D4}$of the DEC_C_ can be obtained:

$V_{D4}=\frac{Q_{D3}}{C_{D4}}=\frac{Q_{D2}+Q_{T}}{C_{D4}}=\frac{C_{Dmin}V_{D1}+Q_{T}}{C_{D4}}$ (3-15)

${V_{D4}=V}_{D3}+\frac{V_{D_{3}}}{2}=\frac{{3V}_{D_{3}}}{2}=\frac{Q_{s4}(C_{s}{+C}_{M})}{2C_{s}C_{M}}$ (3-16)

As the charges of the DEC_C_ in two points (3 and 4) are equal, the capacitance $C_{D4}$of DEC_C_ can be expressed:

$C_{D4}=\frac{V_{D3}C_{Dmax}}{V_{D4}}$ (3-17)

According to Equation (3-12), at point 4, the total charge $Q_{s4}$ in the charge pump circuit can be described:

$Q_{s4}=\frac{3C_{s}C_{M}V_{D3}}{C_{s}+C_{M}}= \frac{3C_{s}C_{M}(Q_{D2}+Q_{T})}{(C_{s}+C_{M}{)C}_{Dmax}}$ (3-18)

Due to $V_{s4}=V_{D4}$, the voltage $V_{s4}$ of the charge pump circuit can be written:

$V_{s4}=V_{D4}=\frac{2Q_{s4}}{C_{0}}$ (3-19)

According to Equations (3-17) and (3-19), the charge $Q_{S4}$ in the charge pump circuit can be written:

$Q_{S4}=C_{Dmin}V_{D1}+Q_{T}$ (3-20)

By combining Equations (3-20) and (3-12), the total charge $Q^{4}$in the single-modal generator is given by:

$Q^{4}=Q_{S4}+Q_{D4}=\frac{3C_{s}C_{M}(Q_{D2}+Q_{T})}{(C_{s}+C_{M}{)C}_{Dmax}}+C_{Dmin}V_{D1}+Q_{T}$ (3-21)

4-5: The voltage across the DEC_C_ continuously increases and rises above a certain threshold, allowing the partial charges stored in the DEC_C_ flow to the charge pump circuit. Finally, at the point 5, the DEC_C_ returns to its initial state, and the total amount of charge stored in the single-modal generator increases during the stretching-releasing process.

At point 5, the charges in the charge pump circuit and DEC_C_ are, respectively:

$Q_{S5}=Q_{S4}+Q_{T}^{'}$ (3-22)

$Q_{D5}=Q_{D4}-Q_{T}^{'}$ (3-23)

where $Q_{T}^{'}$ is the shuttling charge during the process from point 4 to point 5.

Due to $V_{D5}=V_{S5}$, according to Equations (3-22) and (3-23), the shuttling charge from the DEC_C_ to the charge pump circuit can be obtained as follows:

$\frac{Q_{D4}-Q_{T}^{'}}{C_{Dmin}}=$ $\frac{Q_{S4}+Q_{T}^{'}}{C_{SS}}$ (3-24)

$Q_{T}^{'}=\frac{C_{SS}{Q_{D4}-C_{Dmin}Q}_{S4}}{C_{SS}+C_{Dmin}}$ (3-25)

$Q_{D4}$ and $Q_{S4}$ can be substituted by Equation (3-18) and (3-20):

$Q_{T}^{'}=\frac{2C_{S}C_{M}C_{Dmax}\left( C_{Dmin}V_{D1}+Q_{T} \right)-3C_{s}C_{M}C_{Dmin}\left( Q_{D2}+Q_{T} \right)}{2C_{S}C_{M}C_{Dmax}+C_{Dmin}(C_{s}+C_{M}{)C}_{Dmax}}$ (3-26)

$Q_{T}^{'}=\frac{\left( 2n-3 \right)C_{s}C_{M}\left( Q_{D5}+Q_{T}^{'} \right)}{2nC_{S}C_{M}+C_{Dmax}(C_{s}+C_{M})}$ (3-27)

According to Equation (3-15), we can obtain:

$Q_{T}^{'}=\frac{\left( 2n-3 \right)C_{s}C_{M}C_{Dmin}V_{D5}}{3C_{s}C_{M}+C_{Dmax}\left( C_{s}+C_{M} \right)}$ (3-28)

$\frac{V_{D5}}{V_{D1}}=\frac{V_{D5}C_{Dmin}}{V_{D1}C_{Dmin}}=\frac{Q_{D5}}{Q_{D1}}$ (3-29)

There is no charge increase anywhere in the single-modal generator during the process from 4 to 5; thus, the charge is the same at point 5 at 4:

$Q^{5}=Q^{4}=Q_{D4}+Q_{S4}=Q_{D3}+Q_{S4}$ (3-30)

Substituting Equation (3-11) and Equation (3-21) into the Equation (3-27), gives

$Q^{5}=V_{D1}C_{Dmin}+Q_{T}+\frac{3C_{s}C_{M}(Q_{D2}+Q_{T})}{(C_{s}+C_{M}{)C}_{\mathrm{Dmax}}}$ (3-31)

Inserting Equation (3-15) into Equation (3-28) leads to:

$\frac{V_{D5}}{V_{D1}}=\frac{Q^{5}}{Q^{1}}=\frac{C_{Dmax}{(V}_{D1}C_{Dmin}+Q_{T})\left( C_{s}+C_{M} \right)+3C_{s}C_{M}(Q_{D2}+Q_{T})}{C_{Dmax}\left( C_{S}+C_{M} \right)C_{Dmin}V_{D1}+2{V_{D1}C}_{S}C_{M} C_{Dmax}}$ (3-32)

After a stretching-releasing cycle, the charge increased $\Delta Q$ in the single-modal generator can be expressed:

$\Delta Q=Q^{5}-Q^{1}=\frac{{C_{Dmax}Q}_{T}(C_{S}+C_{M}+3C_{s}C_{M})+C_{s}C_{M}(3Q_{D2}-2V_{D1}C_{Dmax})}{\left( C_{S}+C_{M} \right)C_{Dmax}}$ (3-33)

$Q_{T}=C_{Dmin}V_{D1}(\frac{C_{Dmin}}{C_{Dmin}+\frac{{2C}_{s}}{n}+\frac{C_{M}}{2n}}+\frac{3C_{M}C_{S}}{{(C}_{M}+C_{S})(C_{Dmin}+\frac{{2C}_{s}}{n}+\frac{C_{M}}{2n})}-1)$ (3-34)

When $m=\frac{C_{S}}{C_{Dmin}},r=\frac{C_{S}}{C_{M}}=0.5$, and $n=\frac{C_{Dmax}}{C_{Dmin}}$, Equations (3-32), (3-34) can be rewritten as:

$Q_{T}={C_{Dmin}V_{D1}\left( \frac{n\left( 3m+r+1 \right)}{\left( r+1 \right)\left( n+2m+\frac{m}{2r} \right)}-1 \right)}$ (3-35)

${\frac{V_{D5}}{V_{D1}}=\frac{(3r+m+\frac{r}{m})(3r+nm+n\frac{r}{m})}{(2r+m+\frac{r}{m})(n+2m+\frac{r}{2m})(m+\frac{r}{m})}}$ (3-36)

$Q_{T}{=\frac{C_{s}C_{D_{min}}V_{D_{1}}(2n-3)}{nC_{D_{min}}+3C_{s}}}$ (3-37)

${\frac{V_{D5}}{V_{D1}}=\frac{(1.5+m+\frac{1}{2m})(3r+nm+\frac{n}{2m})}{(2r+m+\frac{1}{2m})(n+2m+\frac{1}{4m})(m+\frac{1}{2m})}=\frac{(2m^{2}+2.5)(3r+2nm^{2}+n)}{(2+2m^{2})(n+8m^{2}+1)(2m^{2}+1)}}$ (3-38)

As both $Q_{T}$ and $Q_{2}$ repsent transferred charge between the circuit and the DEC, the transferred charge $Q_{2}$ can be written as:

$Q_{2}=Q_{T}={C_{Dmin}V_{D1}\left( \frac{n\left( 3m+r+1 \right)}{\left( r+1 \right)\left( n+2m+\frac{m}{2r} \right)}-1 \right)}=\frac{C_{S}C_{Dmin}V_{H}(2n-3)}{nC_{Dmin}+3C_{S}}$ (3-39)

In the single-membrane structure under high voltage, when electrostatic stress exceeds the elastic stress of the DE membrane, the material cannot generate sufficient resistance to the excessive squeezing force, resulting in thinning of the DE membrane. As a result, the minimum capacitance $C_{cmin(Single)}$ of the DEC_C_ during operation is higher than its initial capacitance $C_{Cinitial}$ in the uncharged stage, leading to a reduced capacitance change ratio (*n*) during operation. In contrast, DEC_C_ undergoes a decrease in capacitance and an increase in voltage during the release process, creating an electric potential gradient with DEC_A_. This potential difference drives charge transfer from DEC_C_ to DEC_A_, thereby reducing the electrostatic stress on the DEC_C_ compared to the single-membrane configuration. Taking into account the electrostatic stress concentration in the single-membrane structure, the minimum capacitance $C_{cmin(Dual)}$ of the DEC_C_ in the dual-membrane structure is smaller than the minimum capacitance $C_{cmin(Single)}$ of the DEC_C_ in the single-membrane structure. Thus, under the same voltage, the relationship between the minimum capacitance of the DEC_C_ in the single-membrane structure and the dual-membrane structure is

$C_{cmin(Single)}˃ C_{cmin(Dual)}{\geq C}_{cinitial}$ (3-40)

Through equation $V=Q/C$, at the capacitance and its storage charge decrease simultaneously, the voltage remains constant. Meanwhile, the capacitance ($C_{D}$) and capacitance change ratio (*n*) of dual-membrane structure can be expressed:

$C_{Dmin}=C_{Cmin}+C_{Amin}$ (3-41)

$n=\frac{C_{Dmax}}{C_{Dmin}}=\frac{C_{Cmax}+C_{Amin}}{C_{Cmin}+C_{Amax}}$ (3-42)

where $C_{C}$ and $C_{A}$ are the capacitances of DEC_C_ and DEC_A_, respectively. The relation of the $C_{Dmin}$ and $n$ between the single-membrane structure and dual-membrane structure can be obtained:

$C_{Dmin(Single)}=C_{Cmin}<C_{Dmin(Dual)}=C_{Cmin}+C_{Amin}$ (3-43)

$n_{(Single)}=\frac{C_{Cmax}}{C_{Cmin}}>n_{(Dual)}=\frac{C_{Cmax}+C_{Amin}}{C_{Cmin}+C_{Amax}}$ (3-44)

All other things being equal, we can find that the theoretical transferred charge is constant for the $C_{Dmin}$ and $n$ varies within a certain range based on equation 3-39 (**Figure S14, Supporting Information**).

**Supplementary Note 4:**

**Maxwell stress:**

The Maxwell stress ($P$) is generated by the electrostatic attraction between two compliant electrodes, which can be expressed:

$p=\frac{\sigma^{2}}{\varepsilon_{0}\varepsilon}$ (4-1)

where $\sigma$ is the surficial charge density of the DEC_A_; $\varepsilon_{0}$ is vacuum dielectric constant; $\varepsilon$ is the dielectric constant of the dielectric elastomer.

**Calculation for dynamic capacitance change of DEC_A_:**

During the DEC_A_ dynamic deformation, the initial capacitance of DEC_A_ can be expressed as $C_{Ainitial}=\frac{\varepsilon A}{d}$, the capacitance of DEC_A_ at point 1 can be expressed as $C_{A1}=\frac{\varepsilon A_{1}}{d_{1}}$, and the capacitance of DEC_A_ at point 3 can be expressed as $C_{A3}=\frac{\varepsilon A_{3}}{d_{3}}$. Assuming incompressibility of DE materials, $Ad=A_{3}d_{3}=A_{1}d_{1}$, $k_{d}=\frac{A_{1}-A_{3}}{A_{3}}$, $k_{s}=\frac{A_{3}-A}{A}$, the dynamic capacitance change of DEC_A_ ($\Delta C$) can be expressed as:

$\Delta C=C_{A1}-C_{A3}=C_{A3}\left[ {(1+k_{d})}^{2}-1 \right]$ (4-2)

$C_{A3}=C_{Ainitial}{(1+k_{s})}^{2}$ (4-3)

$k_{s}=\frac{A_{3}-A}{A}$ (4-4)

where $\varepsilon$ is the dielectric constant of the DEC_A_, $A$ is the initial electrode area of the DEC_A,_ $d$ is the initial thickness of the DEC_A,_ $A_{1}$ is the electrode area of the DEC_A_ at point 1, and $d_{1}$ is the thickness of the DEC_A_ at point 1. $A_{3}$ is the electrode area of the DEC_A_ at point 3, and $d_{3}$ is the thickness of the DEC_A_ at point 3, $k_{d}$ is dynamic change in the electrode area of the DEC_A_. $k_{s}$ is static change in the electrode area of the DEC_A_.

**Calculation for peak output power and** **average output power of the system:**

Peak power represents the maximum power that can be achieved during the cyclic current variation. In a resistive AC circuit, peak power can be determined by multiplying the square of the peak current ($I$) by the resistance ($R$), denoted as $P_{peak}$:

$P_{peak}=I^{2}R$ (4-5)

Concurrently, average power indicates the mean power over a specified time interval. Computing average power involves integrating the instantaneous product of voltage and current over a cycle and averaging it over the period ($T$). For resistive AC circuit, since the voltage is in phase with the current, the average power can be simplified to the normalization of the integral of the square of current ($I(t)$) multiplied by the resistance ($R$) over a period of time ($T$), denoted as $P_{average}$:

$P_{average}=\frac{1}{T}\int_{0}^{T} I^{2}(t)Rdt$ (4-6)

This integral can be computed based on the specific waveform of the current.

**Supplementary Note 5:**

The fabrication process of the robot is presented in **Figure S30** (**Supporting Information**), and the as-fabricated robot is placed on the ground with its two feet clinging to it. When a mechanical stimulus is applied on the DEC_C_, the voltage of DEC_C_ decreases, and a voltage difference is built between DEC_C_ and DEC_A_, resulting in an outward transfer of charge from the DEC_A_, which in turn induces a contraction in DEC_A_. Correspondingly, under the action of one-way wheel, the robot hindfoot moves forward during the body contraction. The forward movement is completed when the minimum voltage is achieved at point 3, that is, the maximum contraction of the robot is reached (**Figure S31a**, **Supporting Information**). In turn, once the mechanical stimulus is removed on the DEC_C_, the robot forefoot moves forward, meanwhile the hindfoot is locked, during the body expansion until reaching the maximum voltage at point 5 (**Figure S31b**, **Supporting Information**). Thus, the robot moves forward by alternating forward motion of the forefoot and hindfoot during the periodic mechanical stimulation.


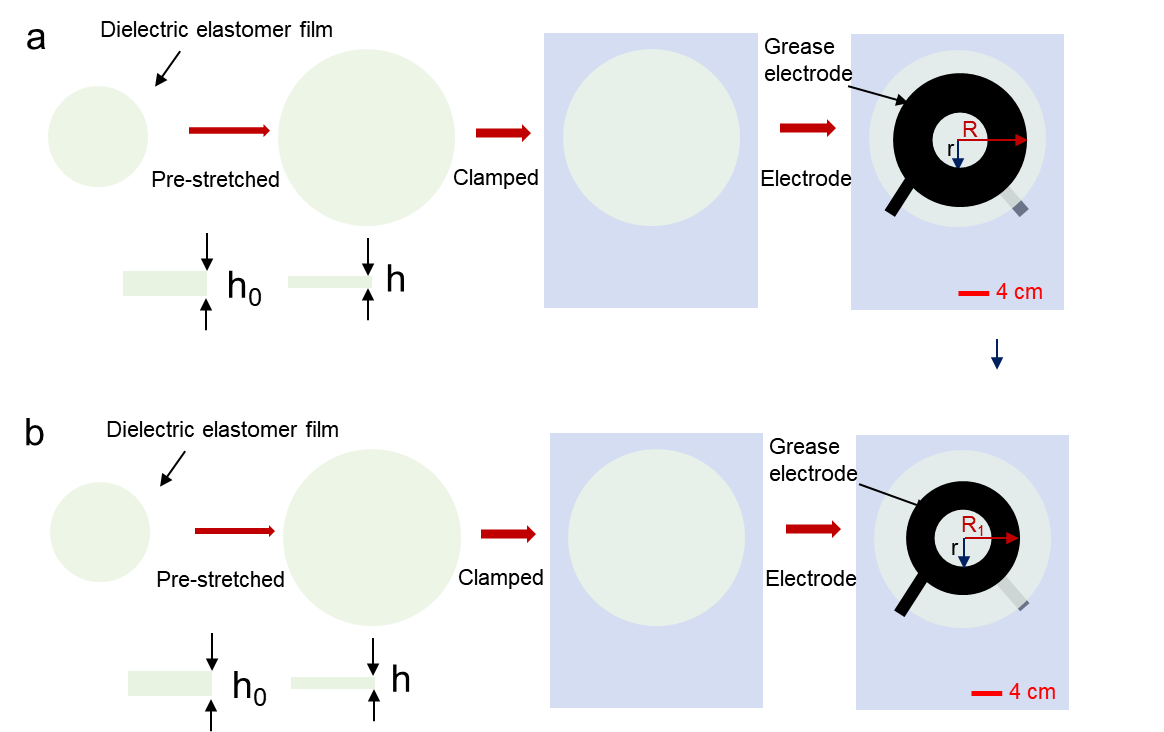


Figure S1 Schematic diagrams of fabrication process of the DEC_C_ (a) and DEC_A_ (b).


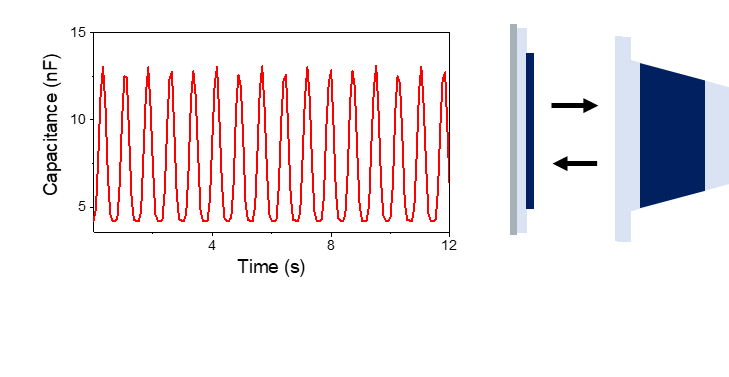


**Figure S2 Dynamic capacitance variations of DEC_C_ under cyclic test at 0.8 Hz.**


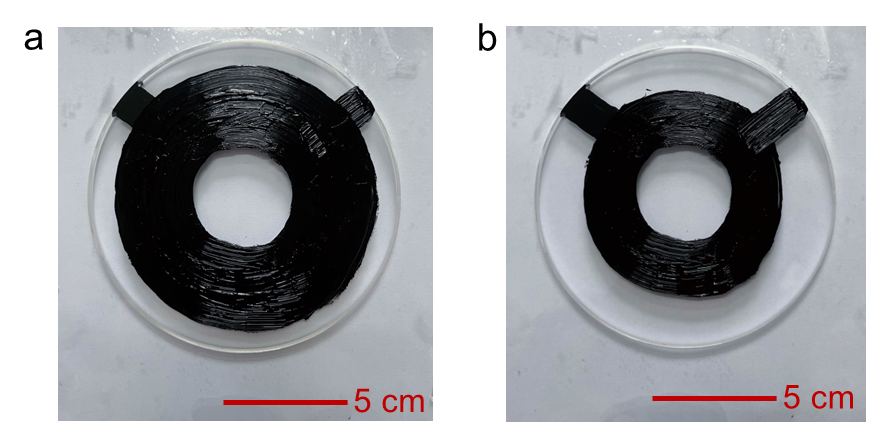


**Figure S3 Photo** **images of the as-fabricated DEC_C_ (a) and DEC_A_ (b).**


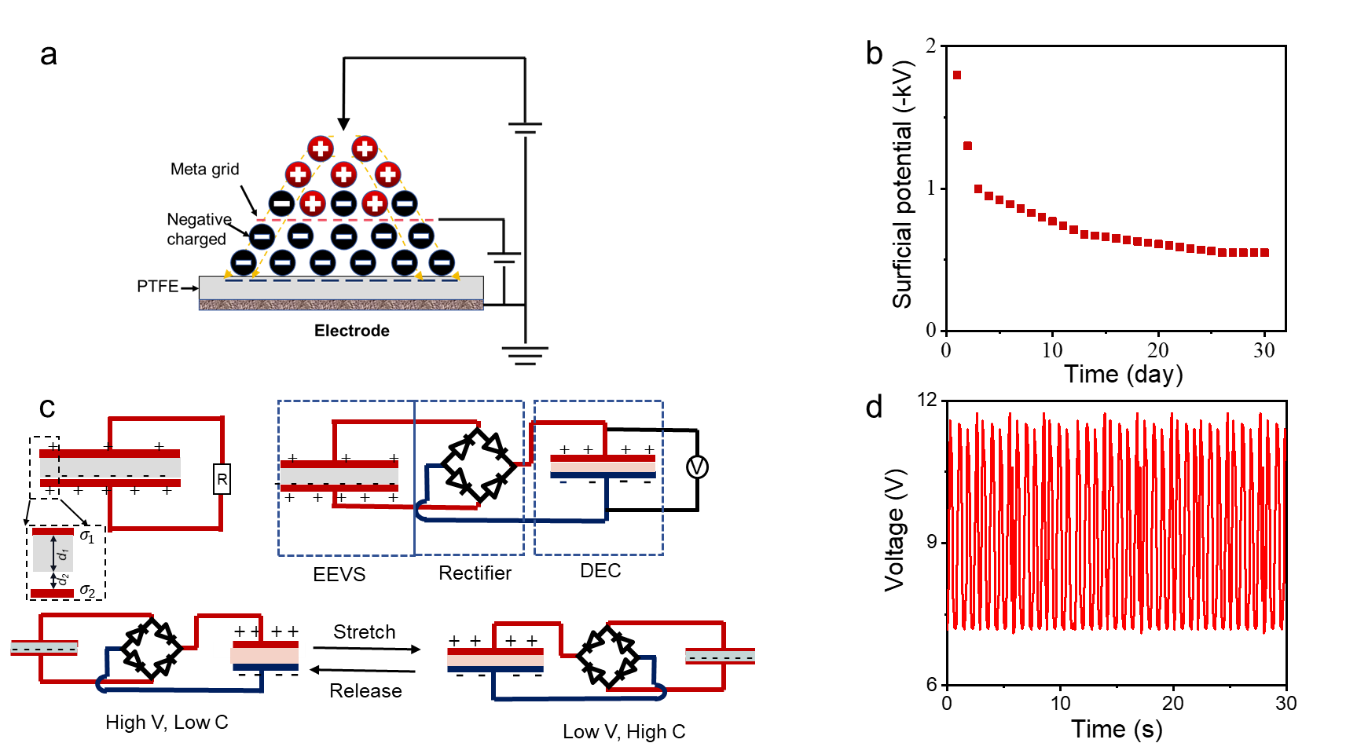


**Figure S4 Fabrication process and electric characteristics of the EEVS. a** Scheme of the corona discharging. **b** Surface potential decay of the electret PTFE film with negative charges. **c** Schematic diagram of voltage testing setup for the DEC. **d** Dynamic voltage of the DEC under 0.8 Hz stretching.


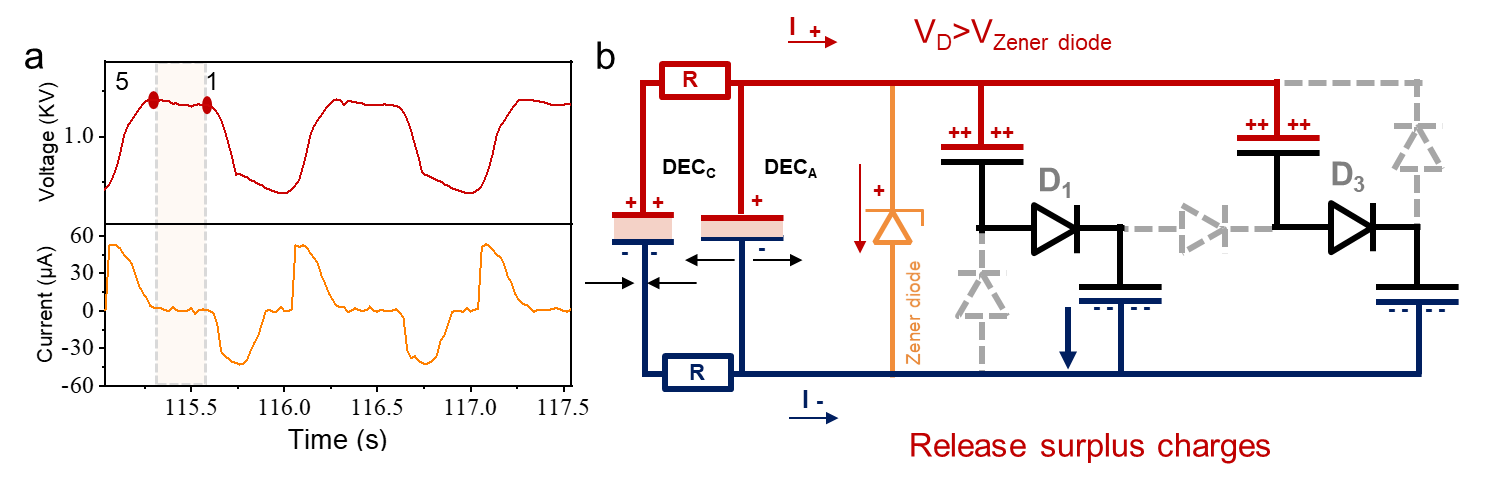


**Figure S5 Work principle of voltage stabilization. a** Dynamic current and voltage with voltage stabilization (1.2 kV) under 0.8 Hz. **b** Schematic diagram of discharge protection at the saturation voltage state.

**
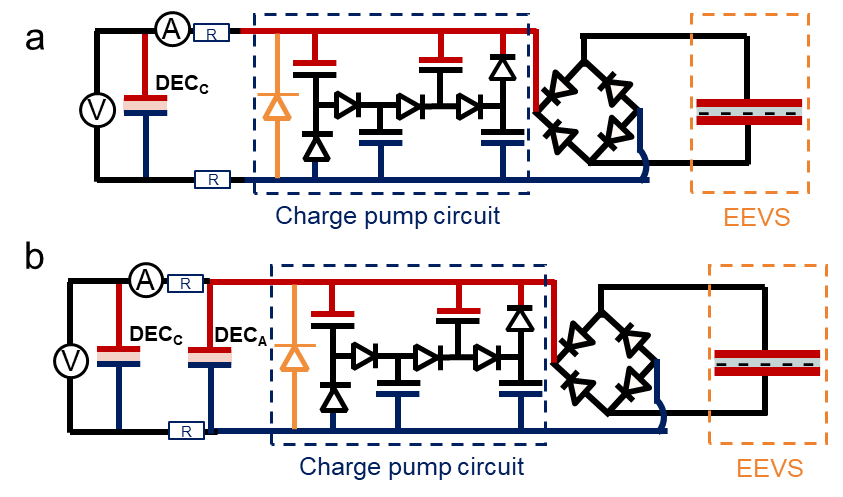
**

**Figure S6** **Schematic diagrams of the voltage and current tests for the Single-modal DE generator (a) and Dual-modal DE system (b).**


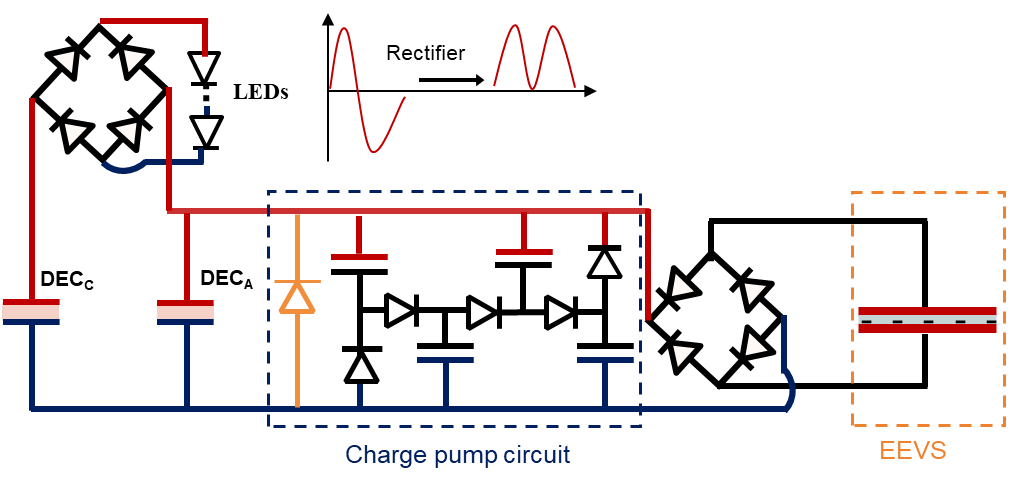


**Figure S7 Schematic diagram of switching from alternating current to direct current via rectifier bridge.**


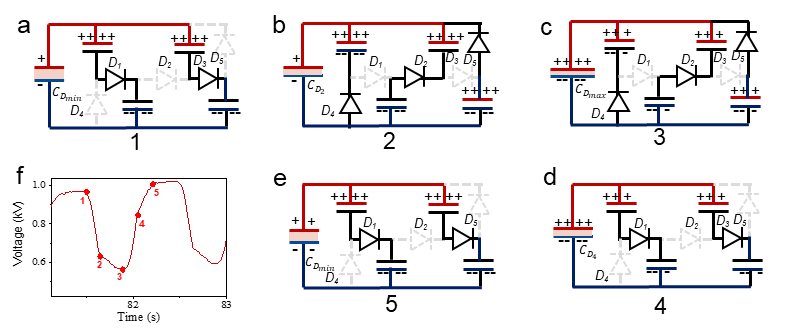


**Figure S8 Schematics diagram of the working mechanism of** **charge pump circuit. a-e** Schematic depicting single-modal DE generator voltage-boost cycle process: Steps 1-3: DEC with stretching; Steps 3-5: DEC with releasing. **f** Schematic depicting voltage-boost cycle process. Steps 2-3: Charges transfer from the circuit to DEC; Steps 4-5: Charges transfer from DEC to the circuit. Point 2: The charge pump circuit switches from series configuration to parallel configuration. The arrangement of diode D_2_ allows for the separation of positive and negative charge carriers (charge generation) by permitting current flow in only one direction. Moreover, it maintains this charge separation state by preventing the flow of charge carriers in the opposite direction (charge recombination). The charge of the charge pump circuit increases after switching the charge pump circuit configuration according to Equation 3-10. The total amount of charge stored in the single-modal generator increases during the stretching-releasing process according to Equation 3-33.

**
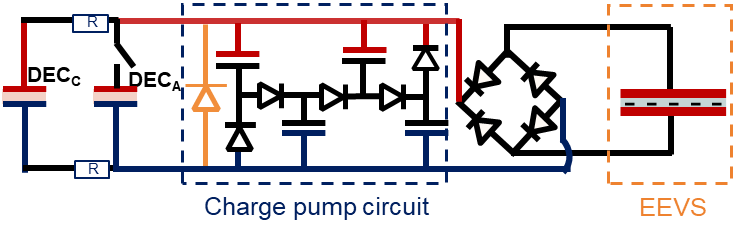
**

**Figure S9 Schematic illustrating the transition mechanism from single-modal DE generator to dual-modal DE system via switch activation.**

**
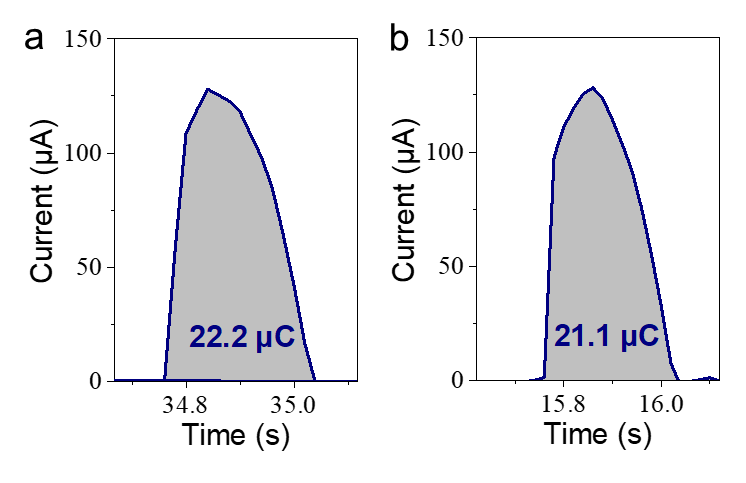
**

**Figure S10** Transferred charge obtained by integration of a short peak current**:** Dual-modal DE system (**a**) **and** Single**-**modal DE generator (**b**).

**
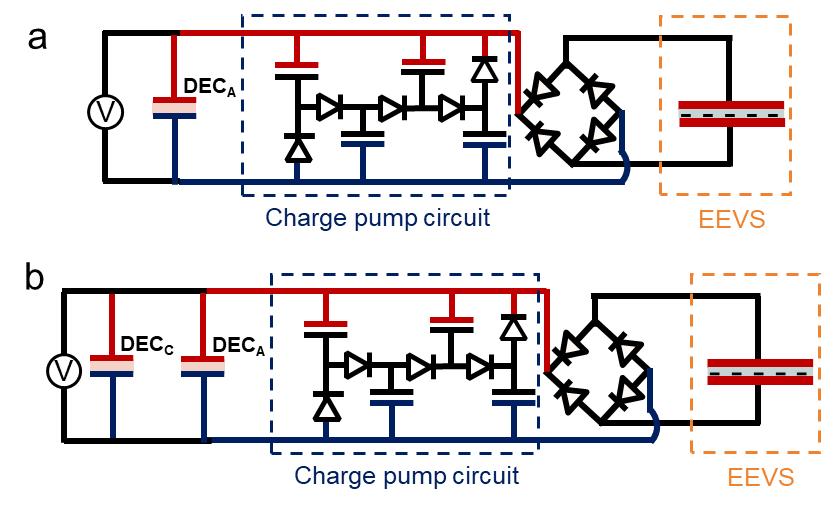
**

**Figure S11 Schematic diagrams of the breakdown voltage tests:** Single**-**modal DE generator (**a**) **and** Dual-modal DE system (**b**).

**
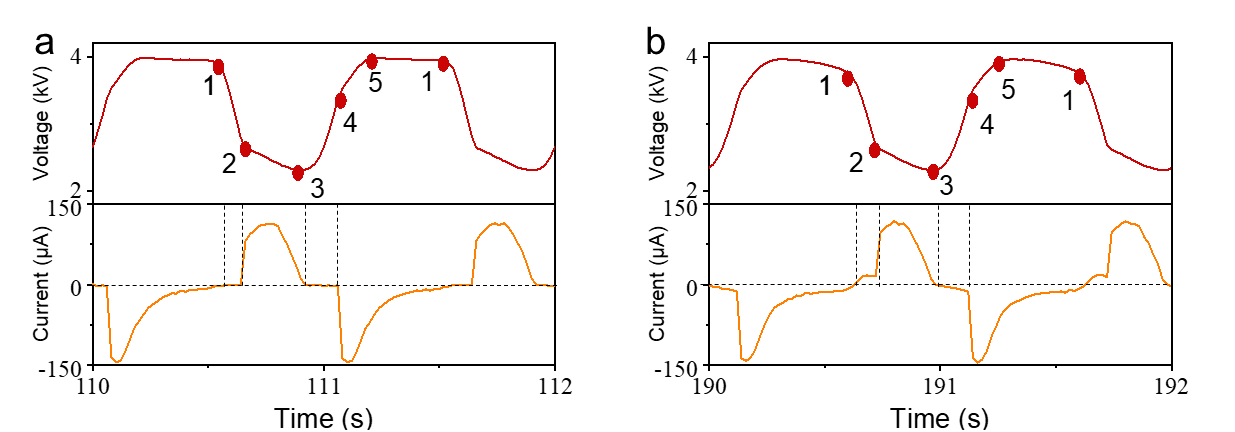
**

**Figure S12 Inflows and outflows of electric charge within the DEC_C_:** Single**-**modal DE generator (**a**) **and** Dual-modal DE system (**b**).

During phase (1-2), when external mechanical stress is applied to the DEC_C_, its capacitance increases and voltage decreases, creating a voltage difference between the DEC_C_ and the charge pump circuit. However, due to the reverse bias of diodes D_1_ and D_3_, no charge transfer occurs in the single-modal DE generator. In contrast, in the dual-modal DE system, the introduction of DEC_A_ enables partial charge transfer from DEC_A_ to DEC_C_ as the voltage of DEC_C_ decreases, maintaining electrical equilibrium between the two.

During phase (3-4), due to the reverse bias of diodes D_1_ and D_3_, no charge transfer occurs in the single-modal DE generator. In the dual-modal DE system, an increase in voltage across the DEC_C_ induces charge transfer from DEC_C_ to DEC_A_.

In phase (5-1), in the single-modal DE generator, DEC_C_ releases charge to the Zener diode to maintain voltage. In the dual-modal DE system, DEC_C_ releases charge to the Zener diode and continues to transfer charge to DEC_A_.

**
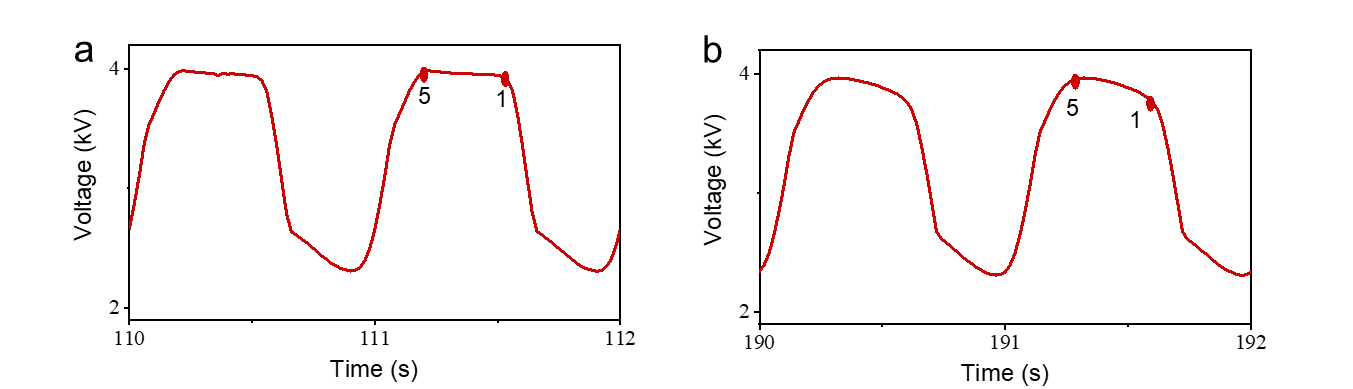
**

**Figure S13 Dynamic voltage of the DEC_C_ during releasing process:** Single**-**modal DE generator (**a**) **and** Dual-modal DE system (**b**).

**Figure S14 Theoretical transferred charge *Q_2_*** **between DEC and circuit. The theoretical results can be obtained by using equation 3-39.**


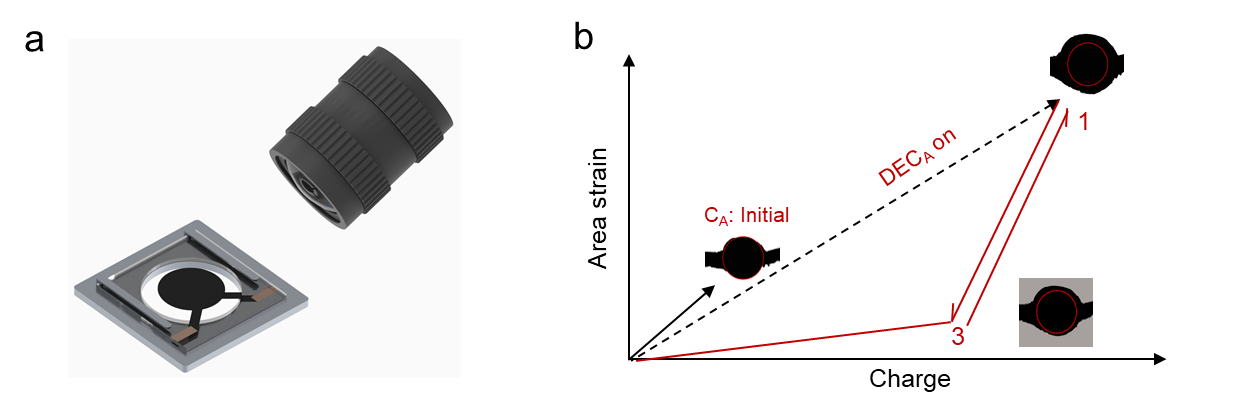


**Figure S15** Area strain schematic of the test configuration for the DEC_A_ (**a**) and photo images of area strain of DEC_A_ during a cycle (**b**).


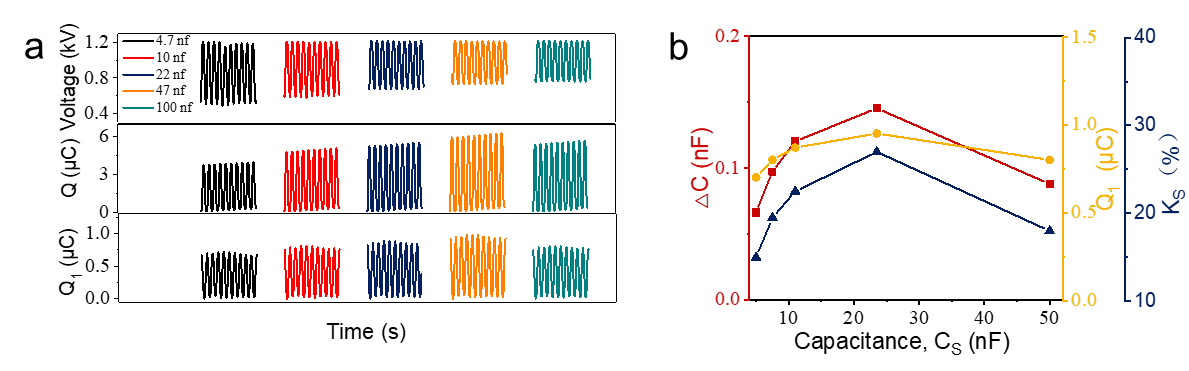


**Figure S16 Effect of different individual capacitor *C_s_* in the pump circuit on the transferred charge of DEC_C_, *Q*, and DEC_A_, *Q_1_*. a** Dynamic voltage and transferred charge of dual-modal DE system. **b** Dynamic capacitance change of DEC_A_, transferred charge to DEC_A_, and static change in the electrode area of the DEC_A_, as described by equation 4-2 and 4-4.

**
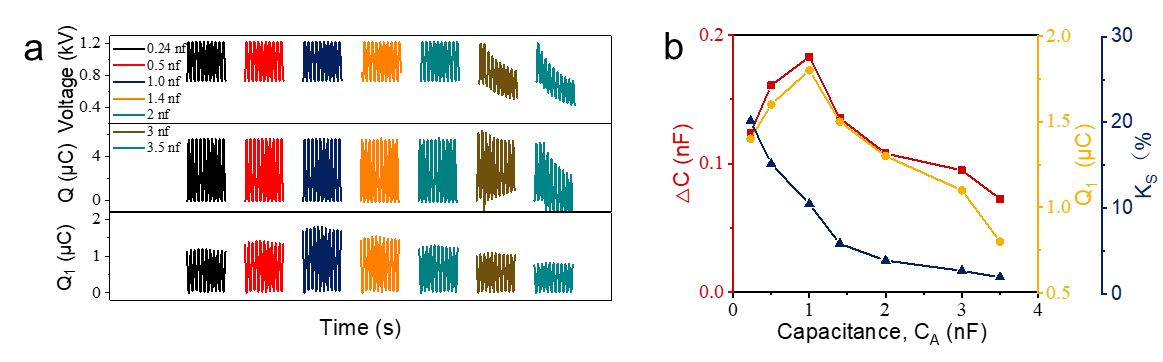
**

**Figure S17 Effect of different capacitance of DEC_A_ on the transferred charge of DEC_C_, *Q*, and DEC_A_, *Q_1_*. a** Dynamic voltage and transferred charge of dual-modal DE system. **b** Dynamic capacitance change of DEC_A_, transferred charge to DEC_A_, and static change in the electrode area of the DEC_A_, as described by equation 4-2 and 4-4.

**
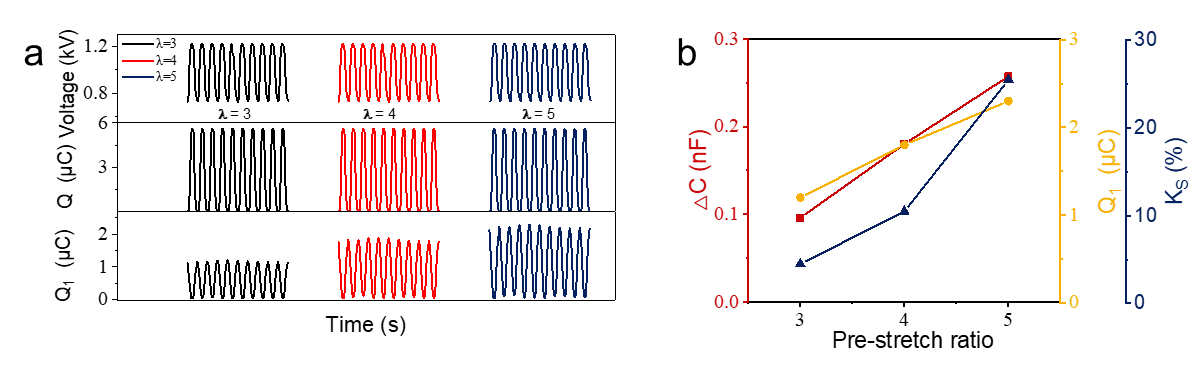
**

**Figure S18 Effect of different pre-stretch ratios of DEC_A_ on the transferred charge of DEC_C_**, ***Q*, and DEC_A_, *Q_1_*. a** Dynamic voltage and transferred charge of dual-modal DE system. **b** Dynamic capacitance change of DEC_A_, transferred charge to DEC_A_, and static change in the electrode area of the DEC_A_, as described by equation 4-2 and 4-4.

**Figure S19. Actuation and electric performance of dual-modal DE system under different humidity.**


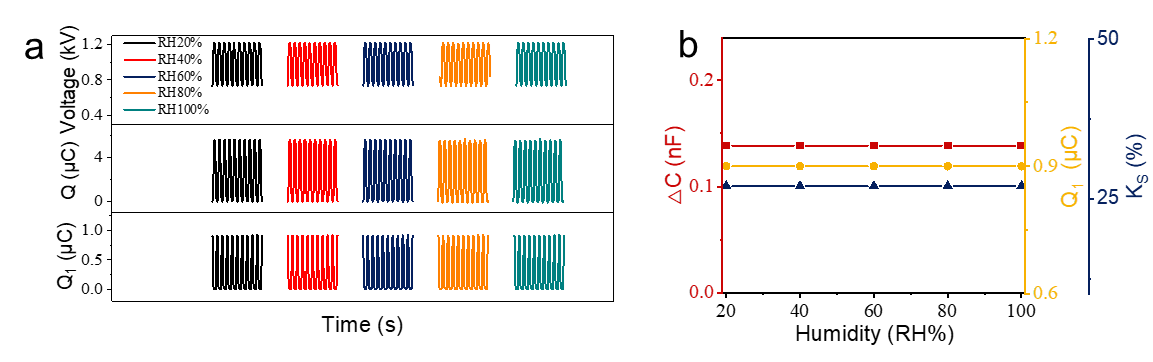


**Figure S20 Effect of different humidity on the transferred charge of DEC_C_, *Q*, and DEC_A_, *Q_1_*. a** Dynamic voltage and transferred charge of dual-modal DE system. **b** Dynamic capacitance change of DEC_A_, transferred charge to DEC_A_, and static change in the electrode area of the DEC_A_, as described by equation 4-2 and 4-4.

**Figure S21** **Actuation and electric performance of dual-modal DE system under different mechanical frequency.**


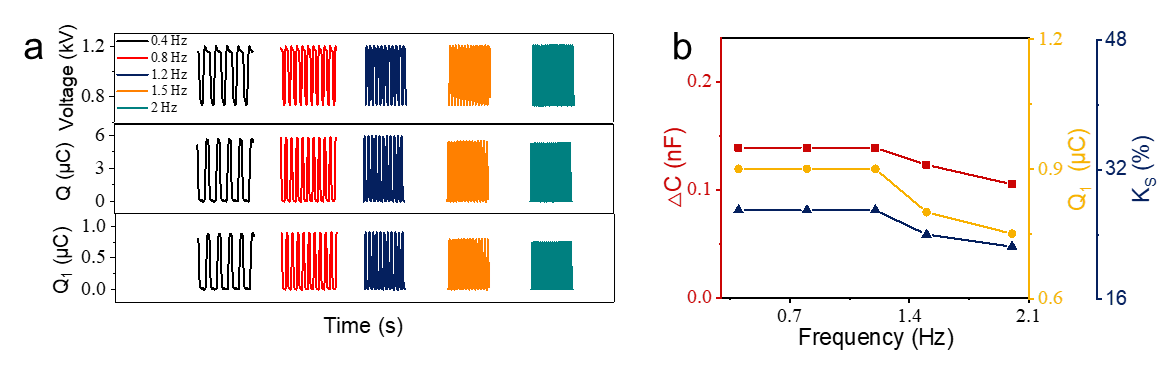


**Figure S22 Effect of different mechanical frequency on the transferred charge of DEC_C_, *Q*, and DEC_A_, *Q_1_*. a** Dynamic voltage and transferred charge of dual-modal DE system. **b** Dynamic capacitance change of DEC_A_, transferred charge to DEC_A_, and static change in the electrode area of the DEC_A_, as described by equation 4-2 and 4-4.

**Figure S23 Actuation and electric performance of dual-modal DE system under different saturating voltage.**


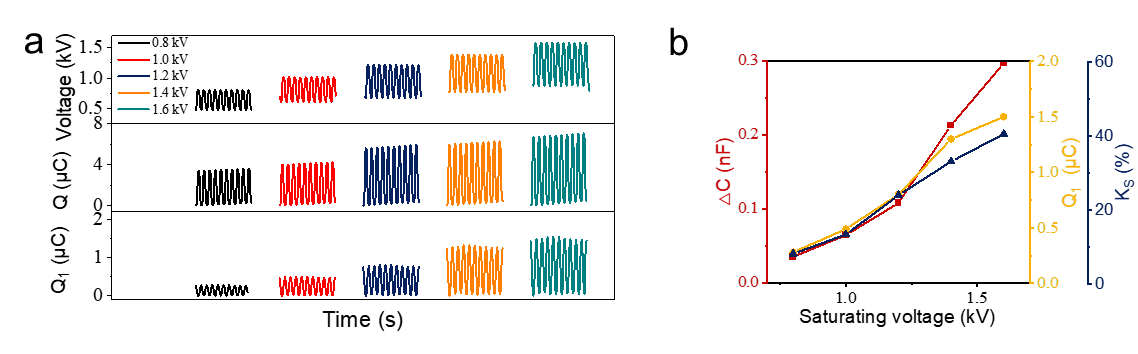


**Figure S24 Effect of different saturation voltage on the transferred charge of DEC_C_, *Q*, and DEC_A_ *Q_1_*. a** Dynamic voltage and transferred charge of dual-modal DE system. **b** Dynamic capacitance change of DEC_A_, transferred charge to DEC_A_, and static change in the electrode area of the DEC_A_, as described by equation 4-2 and 4-4.


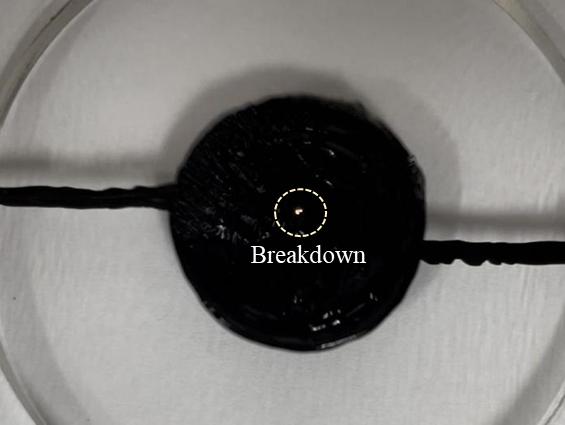


**Figure S25 DEC_A_ breakdown in the dual-modal DE system occurs when *H_A_/H_C_*<1.**


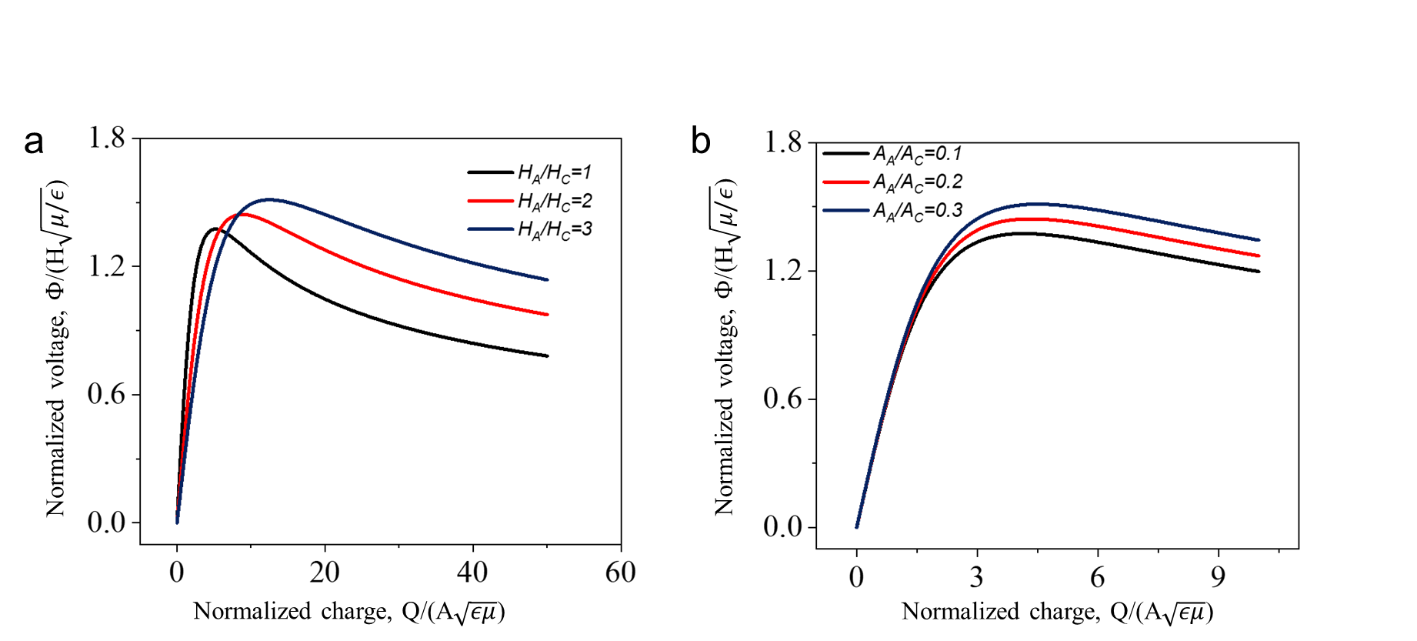


**Figure S26 States of equilibrium plotted on the charge-voltage plane. a** Different thickness ratio (*H_A_/H_C_*). **b** Different area ratio (*A_A_/A_C_*).


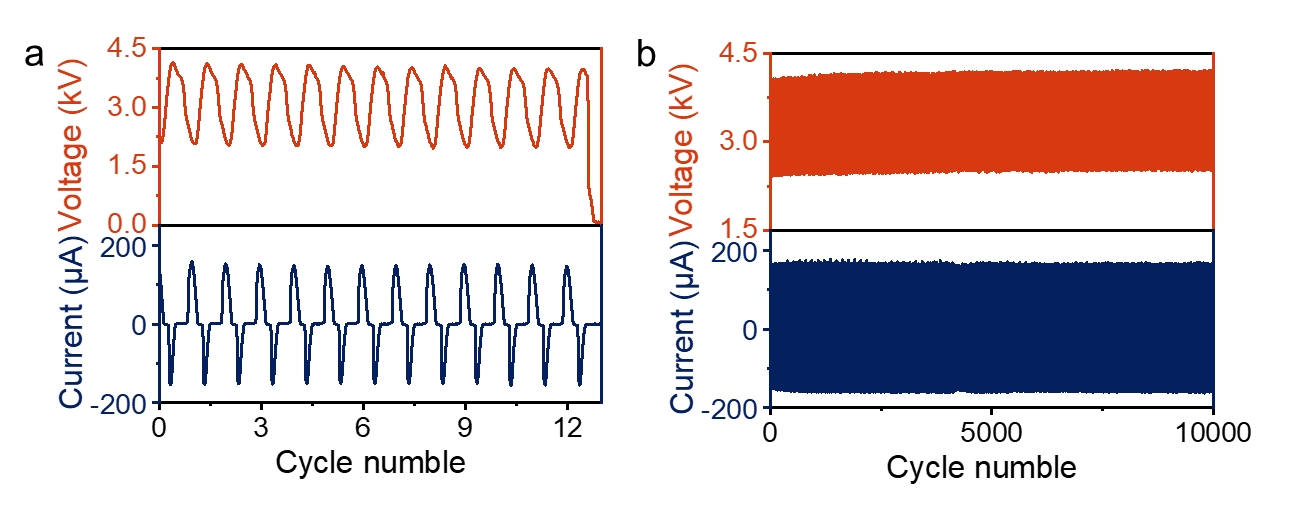


**Figure S27 Fatigue measurements under the saturation voltage of 4.1 kV. a** Single**-**modal DE generator. **b** Dual-modal DE system.


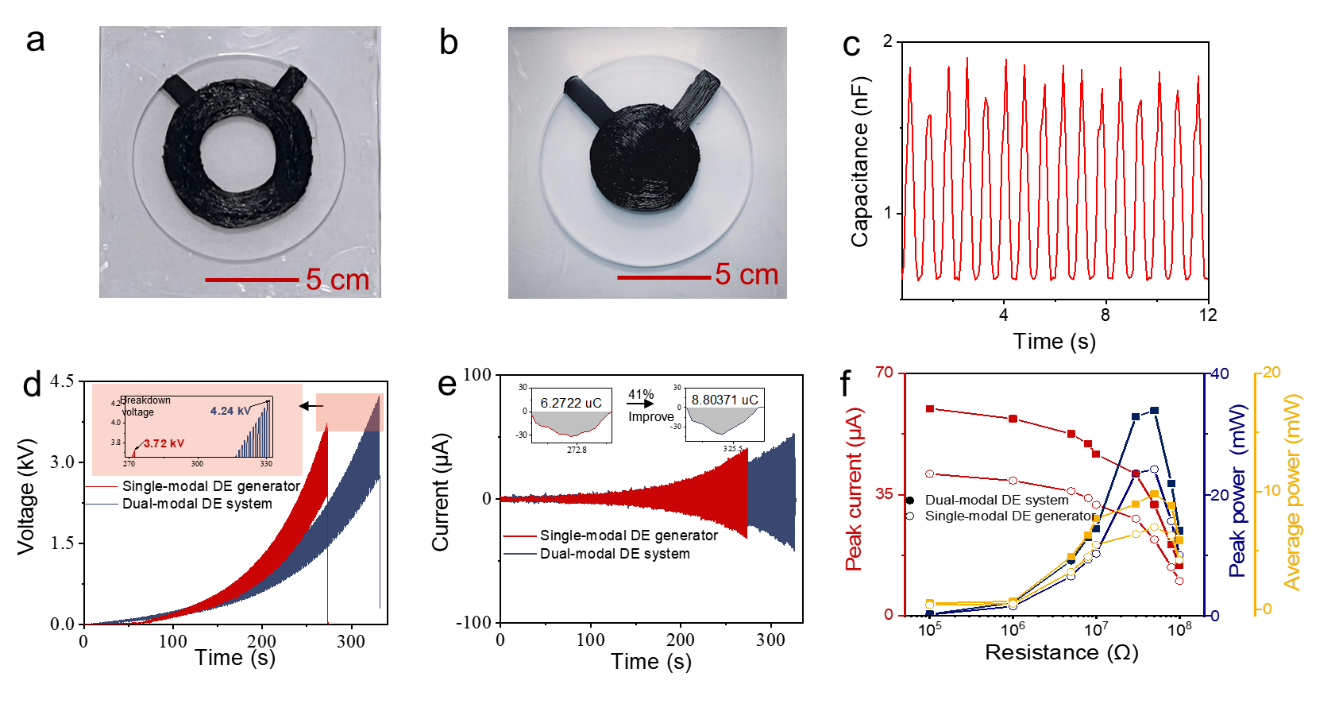


Figure S28 Photo images of as-fabricated silicone-based DEC_C_ (a) and DEC_A_ (b). c Dynamic capacitance variations of DEC_C_ under cyclic test at 0.8 Hz. Comparison of electrical performance of single-modal DE generator and dual-modal DE system (d–e): Breakdown voltage (d); Electric current (e). f Comparison of maximum electric performance achieved by single-modal DE generator and dual-modal DE system. The saturation voltage of the single-modal DE generator and dual-modal DE system is 3.6 kV and 4.1 kV respectively. As a comparison with the single-modal silicone-based DE generator, the dual-modal silicone-based DE system increased transferred charge, peak power, and average power increased from 6.27 μC, 24.20 mW, and 7.01 mW to 8.80 μC, 33.88 mW, and 9.80 mW, respectively. (For the preparation of the silicone materials, Ecoflex 00-30 Part A and Part B (Smooth-On, America) are mixed in a 1:1 mass ratio, ensuring thorough stirring for a homogeneous blend. The mixture is then uniformly applied as a 0.45 mm thick film using an automatic coating machine (MSK-AFA, Kejin, China). The film is then cured in a vacuum drying oven (6020, DZF, China) at 70°C for 1 hour to complete the silicone material production. The silicone material is clamped onto a frame, and two electrodes with a symmetrical structure are coated on both sides using a grease electrode (KS-660, Shin-Etsu, Japan). The initial capacitances of the silicone-based DEC_C_ and DEC_A_ are 0.6 nF and 0.3 nF, respectively.)


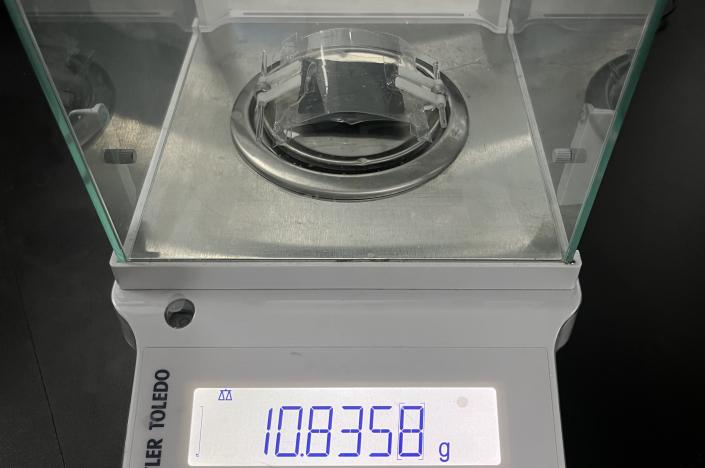


**Figure S29 Photo** **image of soft robot.**


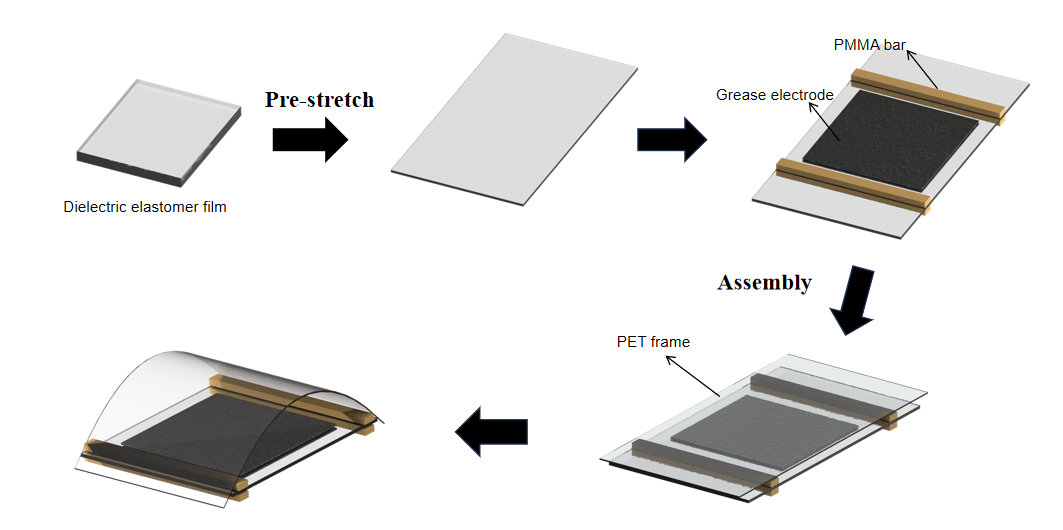


**Figure S30 Fabrication process of the robot.**

**
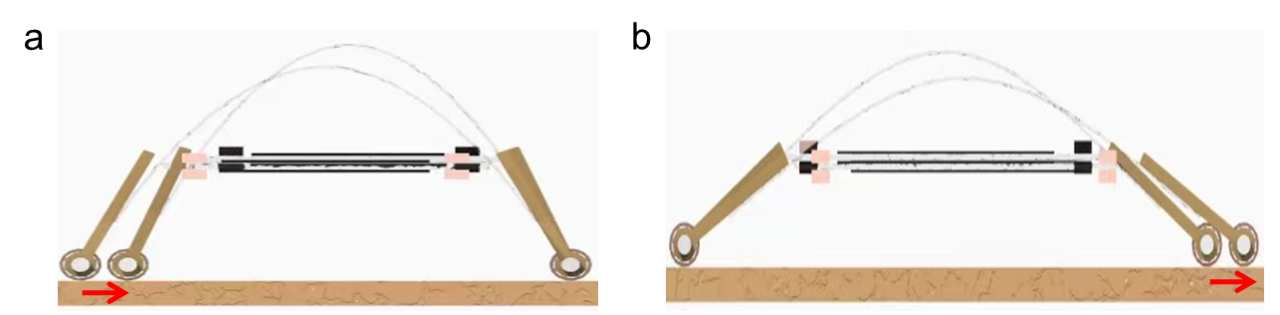
**

**Figure S31 Schematic diagram of the operation of the robot. a** Robot front foot is deactivated and the rear foot is activated immediately. **b** Robot rear foot is deactivated and the front foot is activated immediately.

**
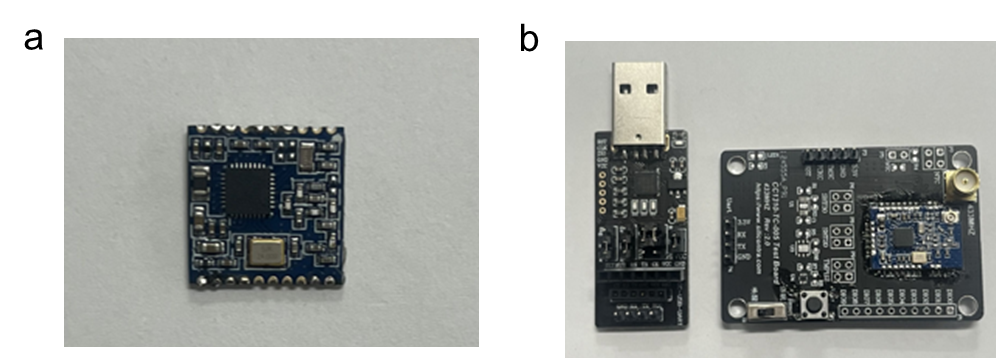
**

**Figure S32 Illustrations of remote temperature reading modules. a** Transmitter. **b** Receiver. Wireless module: CC1310. Temperature measurement module: 433

**Experimental section**

**Preparation of the dual-modal DE system**

The dielectric elastomer (VHB 4905, 3M, America) was pre-stretched radially approximately three times to enhance the stiffness and response speed. Thus, the thickness of the dielectric elastomer became about 80 µm. The thickness was measured using an electronic thickness-measuring and evaluating transducer (C1200, Mahr, Germany). The film was clamped to a frame. Two annular electrodes (DEC_A_ and DEC_C_ both had an inner diameter of 40 mm and outer diameters of 70 and 90 mm, respectively) with a symmetrical structure were coated on the two sides of the dielectric elastomer via a grease electrode (KS-660, Shin-Etsu, Japan). The effective initial area and volume of the DEC_C_ were 20 cm^2^ and 0.16 cm^3^, respectively.

**Characterization of the dual-modal DE system**

The surface potential of the FEP film was measured using an electrometer (Model-541A, Trek, America). The output voltage of the dual-modal DE system was measured using a high-speed electrometer (Model-341, Trek, America). Charges and short currents were measured using with programmable electrometer (6517B and 6514, Keithley, America). The capacitance of the DEC and resistance of the grease were measured using an impedance analyzer (IM3523, HIOKI, Japan). A circular strain test was conducted to measure the actuated strain. During the actuation, video images of the biaxial extension of the electrode area were captured using a camera (Canon, Japan). The strain was defined as the relative change in the pixel of the electrodes' area, which was determined by analyzing the captured video images using Adobe Photoshop software.

**Application of mechanical stress**

Mechanical stress was applied to the DEC_C_ using a crank slider controlled by an actuator motor (86BYG250E, Yixing, China). The operation of the actuator motor was controlled using a motion control system (TC5520, Topcnc, China). The motor generated a sinusoidal displacement with a specified amplitude and frequency, inducing cyclic stretching and releasing of the DEC_C_ membrane.

**Fabrication of the circuit**

The EEVS and other components were electrically connected through a rectifier (KBJ1510). The charge pump circuit was fabricated using five rectifier diodes (2CL71A) and four commercial polyester capacitors (48 nF and 24 nF, type: CBB). A fast recovery diode (GP02-20) was used to stabilize the voltage between the positive and negative pins of the charge pump circuit to avoid dielectric breakdown. Different saturating voltages can be achieved by modulating the combination of the diode GP02-20 and diodes 1N5387B in series.

**Acknowledgments**

**Funding**

This work was financially supported by the National Natural Science Foundation of China (62101498, 52075499), Key Project of Natural Science Foundation of Zhejiang Province (Z24E070008) and Zhejiang Province Public Welfare Technology Application Research Project (LGG22F010005).

**Author contributions**

Z. Xu and S. E designed the research. Z. Zhang and W. Huang carried out the fabrication, characterization, and data analysis. Z. Xu and Z. Zhang wrote the manuscript. S. E guided the project. S. Zheng, J. Tan, J. Cheng, and J. Cai helped with the experiments. All authors reviewed and commented on the manuscript.

**Competing interests**

The authors declare no competing interests.

**Data and materials availability**

All data are available in the main text or supplementary materials. The data that support the findings of this study are available from the corresponding author upon reasonable request.

**Supplementary references**

[1] T. Lu, C. Keplinger, N. Arnold, S. Bauer, Z. Suo, [*Appl. Phys. Lett.*](https://www.x-mol.com/paper/journal/182?r_detail=1409140398905675776) **2014**, *104*, 022905.

[2] T. Lu, C. Ma, T. Wang, [*Extreme Mech. Lett.*](https://www.x-mol.com/paper/journal/7102?r_detail=1261542328497184768) **2020**, *38*, 100752.

[3] Y.Suzuki, *IEEJ Trans. Electr. Electron. Eng.* **2011**, *6*, 101
